# Engineering of Nebulized Metal–Phenolic Capsules for Controlled Pulmonary Deposition

**DOI:** 10.1002/advs.201902650

**Published:** 2020-01-10

**Authors:** Yi Ju, Christina Cortez‐Jugo, Jingqu Chen, Ting‐Yi Wang, Andrew J. Mitchell, Evelyn Tsantikos, Nadja Bertleff‐Zieschang, Yu‐Wei Lin, Jiaying Song, Yizhe Cheng, Srinivas Mettu, Md. Arifur Rahim, Shuaijun Pan, Gyeongwon Yun, Margaret L. Hibbs, Leslie Y. Yeo, Christoph E. Hagemeyer, Frank Caruso

**Affiliations:** ^1^ ARC Centre of Excellence in Convergent Bio‐Nano Science and Technology, and the Department of Chemical Engineering The University of Melbourne Parkville Victoria 3010 Australia; ^2^ Nanobiotechnology Laboratory Australian Centre for Blood Diseases Central Clinical School Monash University Melbourne Victoria 3004 Australia; ^3^ Department of Chemical Engineering Materials Characterisation and Fabrication Platform The University of Melbourne Parkville Victoria 3010 Australia; ^4^ Department of Immunology and Pathology Central Clinical School Monash University Melbourne Victoria 3004 Australia; ^5^ Monash Biomedicine Institute Department of Microbiology Monash University Clayton Victoria 3800 Australia; ^6^ School of Chemistry and the Department of Chemical Engineering The University of Melbourne Parkville Victoria 3010 Australia; ^7^ Micro/Nanophysics Research Laboratory School of Engineering RMIT University Melbourne Victoria 3001 Australia

**Keywords:** aerodynamic diameter, capsules, metal–phenolic networks, nebulization, pulmonary delivery

## Abstract

Particle‐based pulmonary delivery has great potential for delivering inhalable therapeutics for local or systemic applications. The design of particles with enhanced aerodynamic properties can improve lung distribution and deposition, and hence the efficacy of encapsulated inhaled drugs. This study describes the nanoengineering and nebulization of metal–phenolic capsules as pulmonary carriers of small molecule drugs and macromolecular drugs in lung cell lines, a human lung model, and mice. Tuning the aerodynamic diameter by increasing the capsule shell thickness (from ≈100 to 200 nm in increments of ≈50 nm) through repeated film deposition on a sacrificial template allows precise control of capsule deposition in a human lung model, corresponding to a shift from the alveolar region to the bronchi as aerodynamic diameter increases. The capsules are biocompatible and biodegradable, as assessed following intratracheal administration in mice, showing >85% of the capsules in the lung after 20 h, but <4% remaining after 30 days without causing lung inflammation or toxicity. Single‐cell analysis from lung digests using mass cytometry shows association primarily with alveolar macrophages, with >90% of capsules remaining nonassociated with cells. The amenability to nebulization, capacity for loading, tunable aerodynamic properties, high biocompatibility, and biodegradability make these capsules attractive for controlled pulmonary delivery.

## Graphical abstract

The aerodynamic diameters of metal–phenolic capsules are nanoengineered by increasing their shell thickness, which facilitates tailored capsule deposition in a mechanical lung model. The engineered capsules are promising for pulmonary delivery owing to their robustness for nebulization, biocompatibility, biodegradability, and their capacity for cargo loading and surface functionalization.

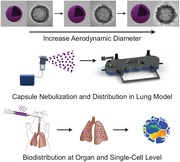

## 1 Introduction

The lung, which is a common site of acute and chronic disease, represents an attractive route for therapeutic administration.^1^ Given its large surface area, high vascularization, and limited metabolic activity,^2^ the deposition of inhaled drugs in the deep lung allows transport of the drugs through the thin epithelial barriers that line the alveolar region so that they can enter the blood circulation system.^3^ Compared to oral administration, pulmonary delivery can achieve a more rapid onset of action, avoiding first‐pass metabolism, and thereby improve the bioavailability of therapeutics.^4^ Alternatively, drug administration via the pulmonary route can allow effective local treatment of lung diseases (e.g., cystic fibrosis, tuberculosis, and lung cancer), as the therapeutic can be directly delivered to the target area.^5^ Such targeted delivery can potentially enhance therapeutic efficacy and hence reduce the dose administered. This in turn can reduce side effects and cost of the treatment.^6^ Moreover, drug delivery to the lungs is noninvasive, thus rendering treatment less obtrusive and more compliant for patients, especially those with chronic diseases, in contrast to needle‐based administration (e.g., intravenous, subcutaneous, or intramuscular injection).

With progress achieved in particle engineering technology, particle‐based pulmonary delivery has emerged as an innovative and promising advance to conventional inhalable dry powder or liquid drug formulations.^7^ Compared with conventional therapeutics, engineered particles can protect the encapsulated drug from degradation,^8^ control the release of drugs over extended periods of time,^9^ and overcome a variety of biological barriers by tailoring the physicochemical properties of the engineered particles.^10^ Some of the biggest challenges in developing particle systems for pulmonary delivery are to maintain colloidal stability (i.e., avoid particle aggregation) during aerosolization and achieve high delivery efficacy.[qv: 7b,11] The aerodynamic diameter (*D*_a_) of the particles is critical for pulmonary delivery, as it determines where the particles are deposited in the respiratory tract following inhalation,^12^ and is defined by

(1) $$D_{a}=\;\;D_{v}\;\sqrt{\frac{\rho }{x\rho_{0}}}$$

where *D*_v_ is the geometric diameter of the particle, ρ is the mass density of the particle, ρ_0_ is the unit density (1 g cm^−3^), and *x* is the dynamic shape factor (1 for a sphere). It is generally accepted that *D*_a_ should be in the range of 1–5 µm for deposition of particles in the deep lung region where they can be therapeutically effective.^13^ Large particles with *D*_a_ > 5 µm will generally deposit in the oropharyngeal region and be ingested,^14^ whereas small particles with *D*_a_ < 1 µm may remain entrained in the airstream and be exhaled during the next breathing cycle.^15^ Thus, controlling *D*_a_ is important, as this allows precise deposition of the particles in specific regions within the lung, thereby improving delivery efficiency, reducing drug exposure to nontargeted regions of the airway, and alleviating any harmful side effects.^16^ De Simone and co‐workers, for example, have demonstrated precise control of *D*_a_ by designing dry powder drug particles with uniform particle shape, size, and morphology using the Particle Replication in nonwetting templates (PRINT) technology.^17^ In addition, variations in size and particle porosity have been investigated in dry powder formulations composed of polymers including poly(lactic acid‐*co*‐glycolic acid).^18^ These studies have involved dry powder aerosols. In contrast, studies investigating the fine control of the aerodynamic behavior of particles with highly defined geometry in liquid aerosols are limited. In nebulized liquid formulations, *D*_a_ is influenced primarily by the nebulizer properties, and particle engineering can provide fine control of the physicochemical properties (e.g., density, size, stiffness, and surface chemistry) of the particles, and hence their aerodynamic properties, in addition to controlling the bio–nano interactions between the drug‐loaded particles in the droplet and the biological environment in which they deposit.

Drug delivery systems based on metal–phenolic networks (MPNs), which are formed upon coordination of metal ions to phenolic compounds, have emerged as promising candidates for biomedicine owing to their distinct properties, including pH responsiveness, high biocompatibility, and high mechanical stability.^19^ MPN‐based, drug‐loaded capsules with highly defined physical properties (e.g., size, shape, and structure) can be obtained through the assembly of MPNs on sacrificial templates,^20^ thus enabling additional degrees of versatility for drug delivery, including the encapsulation of drugs with different physicochemical properties. Varying the phenolic ligands and metal ions also allows the functional properties of the MPN capsules to be tailored for controlled drug release,^21^ cancer cell targeting,^22^ and medical imaging (e.g., positron emission tomography and magnetic resonance imaging).^23^ Nevertheless, the design and study of MPN‐based materials as drug delivery carriers have mainly been limited to delivery via intravenous injection;^24^ their use in pulmonary delivery applications remains unexplored.

**Scheme 1:**
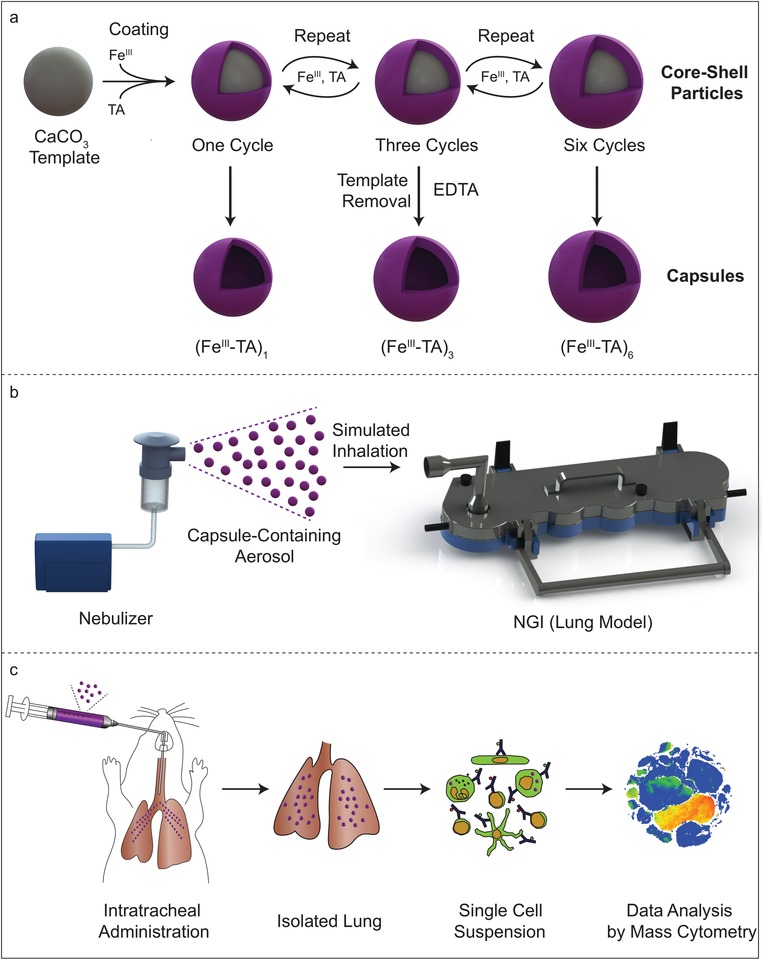
a) Preparation of (Fe^III^‐TA)_1_, (Fe^III^‐TA)_3_, and (Fe^III^‐TA)_6_ capsules with different shell thicknesses, achieved by repeating the MPN coating cycle; the index *n* in (Fe^III^‐TA)*_n_* denotes the number of MPN coating cycles performed. b) Fe^III^‐TA capsule suspensions are aerosolized by an air jet nebulizer and subsequently drawn into the next generation impactor (NGI) to assess airway deposition of the MPN aerosols based on their aerodynamic behavior. c) Fe^III^‐TA capsule suspensions are intratracheally administered into the lungs of a mouse, and 20 h after administration, the lungs are harvested. Single cell suspensions were prepared from the lungs for analysis of cell–capsule interactions by mass cytometry.

In the present study, we nanoengineered MPN capsules with different shell thicknesses through template‐assisted assembly of Fe^III^ and tannic acid (TA) on sacrificial templates (CaCO_3_) and subsequent removal of the templates (**Scheme** 1a). The shell thickness of the capsules was increased by simply repeating the MPN coating cycle. For capsule and aerosol characterization, the capsules were aerosolized using a commercially available air jet nebulizer (Scheme 1b). Particle airway distribution of the nebulized capsules was demonstrated through an in vitro lung model (Scheme 1b) to highlight the advantages of the MPN assembly technique examined herein for facilitating control of pulmonary deposition. Finally, by intratracheal administration of capsules in mice (Scheme 1c), we studied the capsule biodistribution at both the organ and single cell levels, as well as the cellular interactions, biocompatibility, biodegradability, and inflammatory potential of the capsules in the lung.

## 2 Results and Discussion

### 2.1 Assembly and Characterization of MPN Capsules

**Figure 1:**
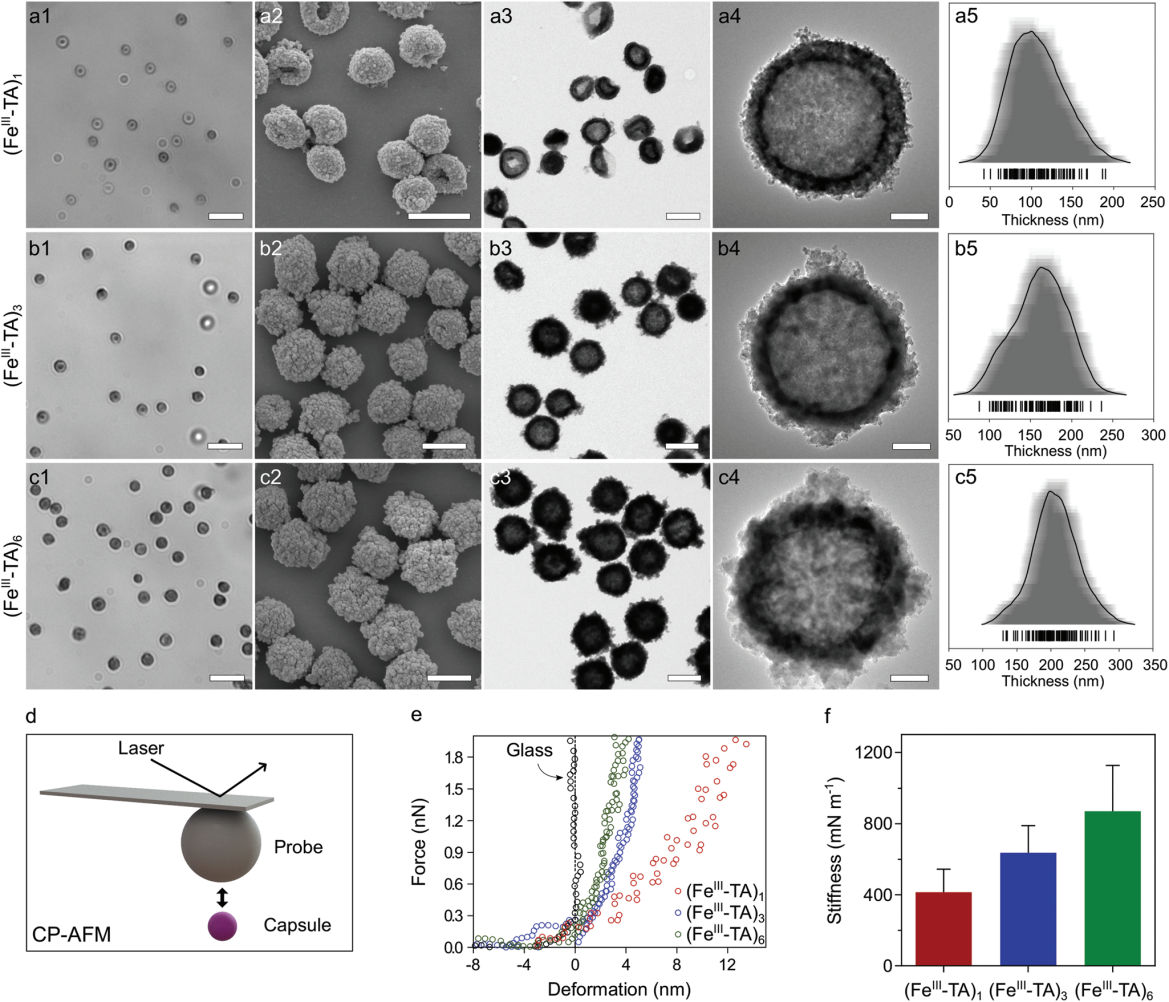
Characterization of the (Fe^III^‐TA)_1_, (Fe^III^‐TA)_3_, and (Fe^III^‐TA)_6_ capsules. a1–c1) Differential interference contrast (DIC) microscopy, a2–c2) scanning electron microscopy (SEM), and a3–c4) transmission electron microscopy (TEM) images of the (Fe^III^‐TA)_1_, (Fe^III^‐TA)_3_, and (Fe^III^‐TA)_6_ capsules. The capsules were suspended in water for DIC microscopy analysis and air‐dried for SEM and TEM analyses. Scale bars are a1–c1) 5 µm, a2–c3) 1 µm, and a4–c4) 200 nm. a5–c5) Shell thickness histograms of the (Fe^III^‐TA)_1_, (Fe^III^‐TA)_3_, and (Fe^III^‐TA)_6_ capsules, respectively, showing mean thicknesses and standard deviations (SDs) of 108 ± 30, 162 ± 31, and 207 ± 32 nm (*n* = 100). d) Schematic representation of the colloidal probe atomic force microscopy (CP‐AFM) technique. e) Representative force–deformation (*F*–δ) curves of the (Fe^III^‐TA)_1_, (Fe^III^‐TA)_3_, and (Fe^III^‐TA)_6_ capsules. The *F*–δ curve of a glass substrate is also shown for comparison. f) Mean stiffness ± SD (*n* = 15) derived from the *F*–δ analysis for the capsules.

To assemble the MPN capsules, CaCO_3_ particles with an average diameter of 1.1 ± 0.3 µm were prepared and used as templates (Figure S1, Supporting Information). Iron(III) chloride hexahydrate (FeCl_3_·6H_2_O) and TA solution were successively added to the suspension of CaCO_3_ particles, followed by the addition of 3‐(*N*‐morpholino)propanesulfonic acid buffer (20 mm, pH 8.5) to increase the pH of the suspension, resulting in the crosslinking of the MPNs owing to the formation of bis‐ and tris‐Fe^III^‐TA complexes.[qv: 19a] TA and Fe^III^ are generally recognized as safe (GRAS) by the U.S. Food and Drug Administration for oral administration. Iron dextran is also used clinically as an intravenous injection to treat iron deficiency.^25^ Following removal of the CaCO_3_ templates using ethylenediaminetetraacetic acid (EDTA) solution (100 mm, pH 7.5) and washing with water, Fe^III^‐TA capsules were obtained (**Figure** 1). The presence of Fe in the capsules and the complete removal of CaCO_3_ and EDTA were confirmed by X‐ray photoelectron spectroscopy (Figure S2, Supporting Information). The shell thickness of the capsules after one TA/Fe^III^ coating cycle (denoted as (Fe^III^‐TA)_1_), was 108 ± 30 nm, which is thicker than typical Fe^III^‐TA films (10 nm)[qv: 19a,23] owing to the adsorption of the Fe^III^‐TA complexes on the highly porous surface of the CaCO_3_ template. By repeating the TA/Fe^III^ coating cycle, the shell thickness gradually increased to 162 ± 31 nm ((Fe^III^‐TA)_3_) and 207 ± 32 nm ((Fe^III^‐TA)_6_), as observed from the transmission electron microscopy (TEM) images (Figure 1a3–c5). This represents an average thickness of ≈20 nm after each MPN coating cycle. The increase in shell thickness was also supported by UV–vis absorption spectroscopy; the intensity of the characteristic ligand‐to‐metal charge transfer band from the Fe^III^‐TA complexes increased as the number of coating cycles increased (Figure S3, Supporting Information). The capsule diameters increased with shell thickness. The diameters of the (Fe^III^‐TA)_1_, (Fe^III^‐TA)_3_, and (Fe^III^‐TA)_6_ capsules were 1.3 ± 0.1, 1.4 ± 0.1, and 1.5 ± 0.1 µm, respectively, in water, as characterized by differential interference contrast microscopy and 0.9 ± 0.1, 1.0 ± 0.1, and 1.1 ± 0.1 µm, respectively, in dry state, as characterized by TEM (Figure S4 and Table S1, Supporting Information). The mass and density of the capsules also increased as more Fe^III^‐TA complexes deposited on the capsule wall with additional cycles (Table S1, Supporting Information).

The (Fe^III^‐TA)_1_, (Fe^III^‐TA)_3_, and (Fe^III^‐TA)_6_ capsules were well dispersed in aqueous solution (Figure 1a1–c1) and remained relatively monodisperse after drying in air (Figure 1a2–c3). The hollow structure, a typical feature of capsules, can be observed from all three capsule systems in the TEM images (Figure 1a4–c4). The (Fe^III^‐TA)_3_ and (Fe^III^‐TA)_6_ capsules did not collapse after air drying, whereas some of the (Fe^III^‐TA)_1_ capsules collapsed and deformed upon air drying (Figure 1a2–c2), suggesting that increasing the shell thickness improved the mechanical properties of the capsules. To explore the mechanical properties of the Fe^III^‐TA capsules, colloidal probe atomic force microscopy measurements were performed on individual (Fe^III^‐TA)_1_, (Fe^III^‐TA)_3_, and (Fe^III^‐TA)_6_ capsules dispersed in aqueous solution on a glass slide (Figure 1d; Figure S5, Supporting Information). The stiffness of the capsules increased with increasing number of coating cycles, as observed in the representative force–deformation curves of each system (Figure 1e). The stiffness, determined from the slope of the curves, increased from 414 ± 130 to 637 ± 153 and 871 ± 258 mN m^−1^ for the (Fe^III^‐TA)_1_, (Fe^III^‐TA)_3_, and (Fe^III^‐TA)_6_ capsules, respectively (Figure 1f). The upward trend suggests a positive relationship between the thickness of the shell and its stiffness, as consistent with previous reports.^26^ The higher stiffness of the (Fe^III^‐TA)_3_ and (Fe^III^‐TA)_6_ capsules relative to that of the (Fe^III^‐TA)_1_ capsules could explain why these capsules retained their spherical shape and did not collapse upon drying.

### 2.2 In Vitro Assessment of Cytotoxicity and Cell Interaction

**Figure 2:**
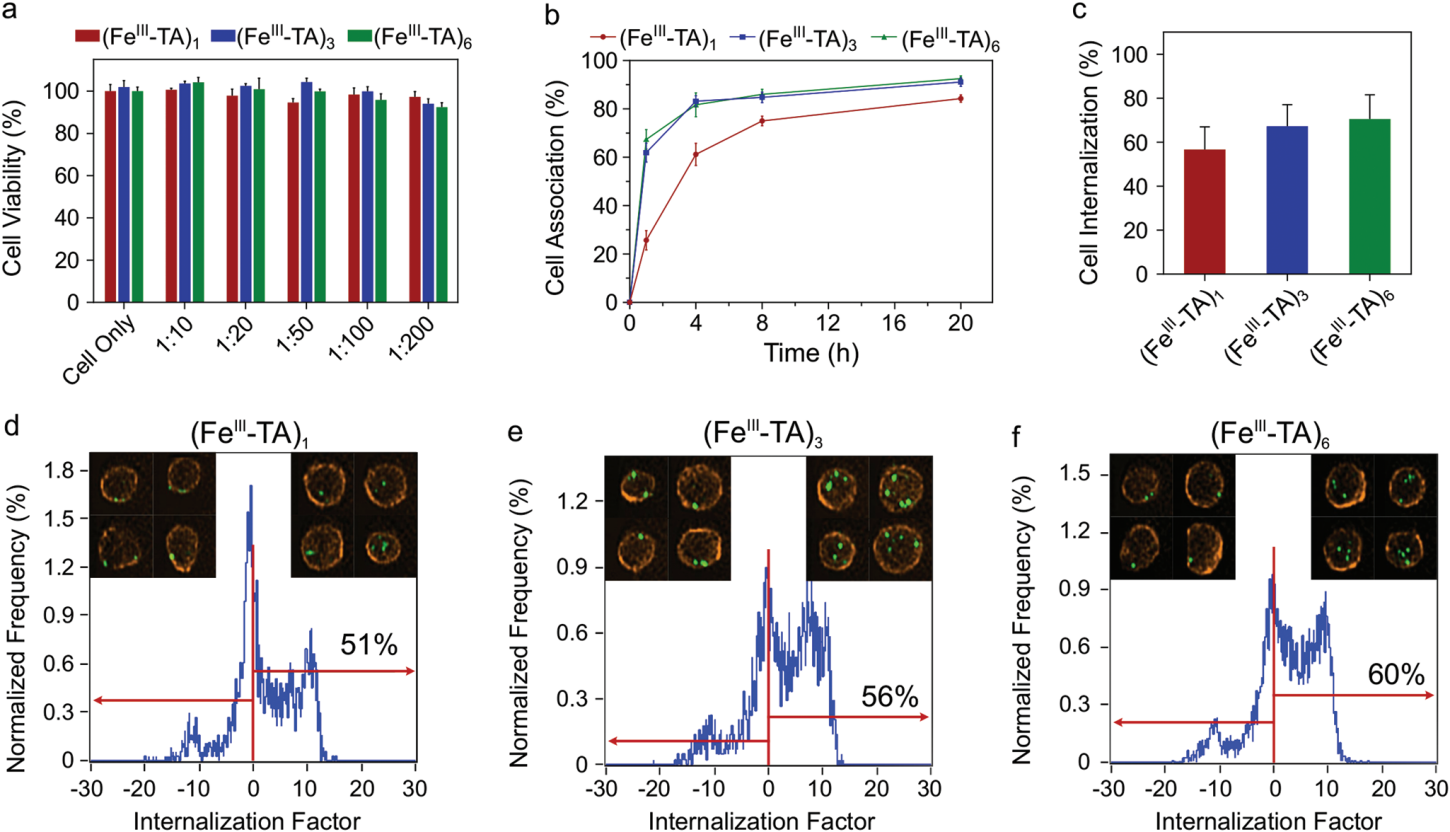
In vitro cell interaction study. a) Cell (A549) cytotoxicity of the (Fe^III^‐TA)_1_, (Fe^III^‐TA)_3_, and (Fe^III^‐TA)_6_ capsules after incubation for 48 h in Dulbecco's modified Eagle's medium with 10% fetal bovine serum at 37 °C at different cell‐to‐capsule ratios. Cell viability was evaluated by the 2,3‐bis[2‐methoxy‐4‐nitro‐5‐sulfophenyl]‐2*H*‐tetrazolium‐5‐carboxyanilide inner salt (XTT) assay (mean ± SD, *n* = 4). The viability of untreated cells was normalized to 100%. b) Association of the (Fe^III^‐TA)_1_, (Fe^III^‐TA)_3_, and (Fe^III^‐TA)_6_ capsules with A549 cells over a 20 h incubation period at 37 °C at a cell‐to‐capsule ratio of 1:100. The percentage of cells that associated with the capsules was determined by flow cytometry (mean ± SD, *n* = 3). c) Internalization of the (Fe^III^‐TA)_1_, (Fe^III^‐TA)_3_, and (Fe^III^‐TA)_6_ capsules in A549 cells after incubation for 20 h at 37 °C at a cell‐to‐capsule ratio of 1:50. The percentage of cells that internalized the capsules was quantified via imaging flow cytometry. No significant differences were observed among (Fe^III^‐TA)_1_, (Fe^III^‐TA)_3_, and (Fe^III^‐TA)_6_ (mean ± SD, *n* = 3, one‐way ANOVA with Tukey's multiple comparisons test). d–f) Representative images showing the quantification of cell internalization via imaging flow cytometry. The capsules were fluorescently labeled by loading with dextran_FITC_ (green), and the cell membranes were stained with Alexa Fluor 594‐wheat germ agglutinin (orange). The degree of cell uptake was expressed as an internalization factor (IF). The insets show the corresponding representative images of cells with externally surface‐bound capsules (negative IF) and cells with internalized capsules (positive IF).

The cytotoxicity of the Fe^III^‐TA capsules with different shell thicknesses was assessed by incubating the capsules with A549 cells at varying cell‐to‐capsule ratios for 48 h. As shown in **Figure** 2a, all three capsule systems had negligible influence on cell viability, even at high capsule dosage. To assess the cell–capsule interactions, the (Fe^III^‐TA)_1_, (Fe^III^‐TA)_3_, and (Fe^III^‐TA)_6_ capsules loaded with dextran–fluorescein (dextran_FITC_, 500 kDa) were incubated with A549 cells for different time periods at 37 °C. Note that the fluorescence signal from the capsules remained stable up to 24 h in cell culture media at 37 °C (Figure S6, Supporting Information). At the early stages of the incubation period, the (Fe^III^‐TA)_3_ and (Fe^III^‐TA)_6_ capsules exhibited faster cell association than the (Fe^III^‐TA)_1_ capsules (>35% difference at 1 h) (Figure 2b). However, this difference became marginal (≈7%) after incubation for 20 h. These findings suggest that shell thickness minimally influences cell association after incubation for 20 h and that the different association kinetics observed for the three capsule systems at the early stages of incubation may be due to the different settling rates of the capsules in aqueous solution (Figure S7, Supporting Information).^27^ Subsequently, the cell internalization of the Fe^III^‐TA capsules was analyzed using imaging flow cytometry. The internalization of the capsules in the cells was quantified by an internalization factor (IF) based on the spatial relationship between the fluorescently labeled capsules and the cell membrane (Figure 2d–f).^28^ A positive IF value correlates to cells that mainly contain internalized capsules, whereas a negative value refers to cells with mainly surface‐bound capsules. As shown in Figure 2c, the percentage of cell internalization (i.e., percentage of cells with positive IFs) increased slightly with increasing capsule coating cycle: 57 ± 10%, 67 ± 10%, and 71 ± 11% for the (Fe^III^‐TA)_1_, (Fe^III^‐TA)_3_, and (Fe^III^‐TA)_6_ capsules, respectively. Consistent with the cell association results, the marginal difference in cell internalization between the different capsule groups suggests that the shell thickness of the Fe^III^‐TA capsules has a minimal influence on cell interactions after 20 h of incubation, given that the different Fe^III^‐TA capsules have a similar surface chemistry with ζ‐potentials ranging from −35 to −40 mV (Table S1, Supporting Information).

### 2.3 Nebulization of Capsules

**Figure 3:**
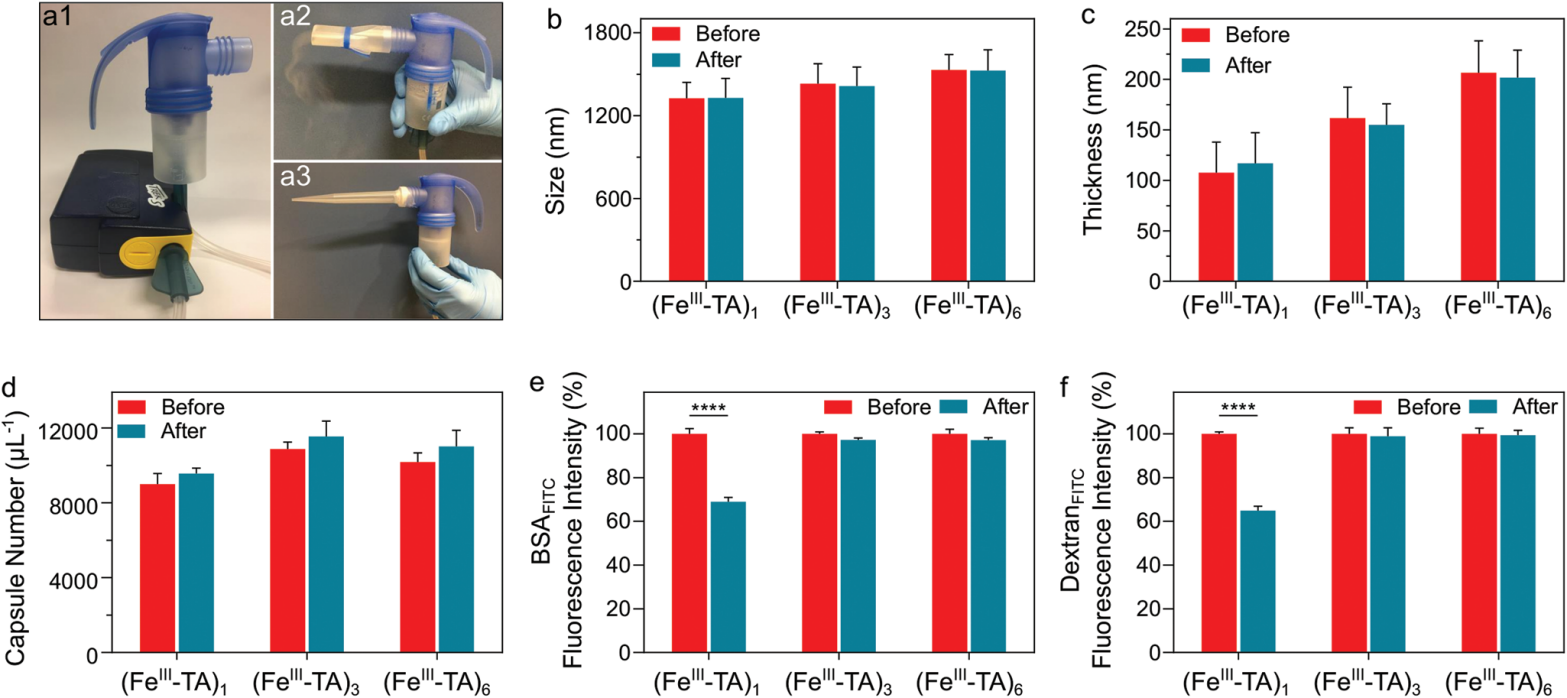
Capsule robustness to nebulization. a1–a3) Photographs of the portable air jet nebulizer used to aerosolize the capsule suspension. The outlet of the nebulizer can be equipped with a2) an adaptor for inhalation or a3) a pipette tip can be attached to the outlet using parafilm to facilitate the collection of the mist as it deposits and accumulates on the walls of the tip for subsequent capsule characterization. b) Size, c) thickness, and d) concentration of the (Fe^III^‐TA)_1_, (Fe^III^‐TA)_3_, and (Fe^III^‐TA)_6_ capsules before and after nebulization. e,f) Fluorescence intensity of the BSA_FITC_‐ and dextran_FITC_‐loaded (Fe^III^‐TA)_1_, (Fe^III^‐TA)_3_, and (Fe^III^‐TA)_6_ capsules before and after nebulization. The fluorescence intensity of the capsules obtained prior to nebulization was normalized to 100% to allow comparison with those obtained following nebulization. The data are presented as the mean ± SD, b) *n* = 30, c) *n* = 100, and d–f) *n* = 3; **** indicates *p* < 0.0001 (two‐way ANOVA with Tukey's multiple comparisons test).

MPN capsules are potentially attractive candidates for pulmonary delivery because of their negligible cytotoxicity, high cargo loading capacity, and tunable physicochemical properties.[qv: 19b,20a] To demonstrate their potential for pulmonary delivery applications, the Fe^III^‐TA capsules were aerosolized using an air jet nebulizer (**Figure** 3a1–a3), which converts a liquid comprising the capsule suspension into a fine inhalable mist (aerosol droplets) using compressed air. For characterization, the nebulized mist containing the capsules was collected using a pipette tip as shown in Figure 3a3 (see section Aerosol Collection, Supporting Information). The collected nebulized capsules were characterized to investigate the robustness of the capsules against the nebulization process. The (Fe^III^‐TA)_1_, (Fe^III^‐TA)_3_, and (Fe^III^‐TA)_6_ capsules did not aggregate and their morphology remained intact after nebulization, as observed from the TEM images (Figure S8, Supporting Information). Specifically, the size and shell thickness of the capsules did not change following nebulization (Figure 3b,c). However, the concentration of the capsules increased slightly by 6–8%, probably due to evaporation of the solvent (water) during the nebulization process (Figure 3d).^29^ To investigate the effect of nebulization on cargo encapsulation, FITC‐labeled bovine serum albumin (BSA) (BSA_FITC_, 65 kDa) and dextran_FITC_ (500 kDa) were used as model drugs and loaded into the (Fe^III^‐TA)_1_, (Fe^III^‐TA)_3_, and (Fe^III^‐TA)_6_ capsules by preadsorption onto porous CaCO_3_ templates prior to Fe^III^‐TA coating. The successful encapsulation of BSA_FITC_ and dextran_FITC_ was confirmed by fluorescence microscopy, which showed homogeneous fluorescence within the capsules regardless of the shell thicknesses (Figures S9 and S10, Supporting Information). After nebulization, the capsules retained their internal fluorescence, indicating that the cargo remained encapsulated. The mean fluorescence intensity of the capsules was measured by flow cytometry to quantify the relative amount of cargo that remained encapsulated after nebulization. A slight decrease (<5%) in the fluorescence intensity of the BSA_FITC_‐ and dextran_FITC_‐loaded (Fe^III^‐TA)_3_ and (Fe^III^‐TA)_6_ capsules was observed after nebulization (Figure 3e,f), indicating minimal leakage of the cargo. In contrast, the fluorescence intensity of the BSA_FITC_‐ and dextran_FITC_‐loaded (Fe^III^‐TA)_1_ capsules after nebulization decreased by 31% and 35%, respectively, suggesting that a greater amount of the cargo was released during nebulization. The release of the cargo may be due to shear forces generated during the nebulization process. These results suggest that the (Fe^III^‐TA)_3_ and (Fe^III^‐TA)_6_ capsules are more robust than the (Fe^III^‐TA)_1_ capsules and therefore have a higher tolerance to the nebulization process, while maintaining cargo encapsulation, probably due to their thicker shell. In addition to protein and macromolecule encapsulation, a small molecule drug, bortezomib (BTZ, 384 g mol^−1^), was conjugated to the Fe^III^‐TA capsules via the formation of pH‐responsive boronic acid–phenol conjugates (Figure S11a, Supporting Information).^30^ The conjugation of BTZ on the capsules was confirmed by energy‐dispersive X‐ray analysis (Figure S11b, Supporting Information). By quantifying the amount of BTZ before and after nebulization using inductively coupled plasma mass spectrometry (ICP‐MS), less than 5% of BTZ was released during nebulization at pH 8.5, thereby confirming the tolerance of drug‐conjugated capsules to nebulization. At alveolar pH (6.6), BTZ was gradually released over 20 h while the capsules remained intact (Figure S12, Supporting Information).

### 2.4 Tuning Capsule Deposition in a Lung Model

**Figure 4:**
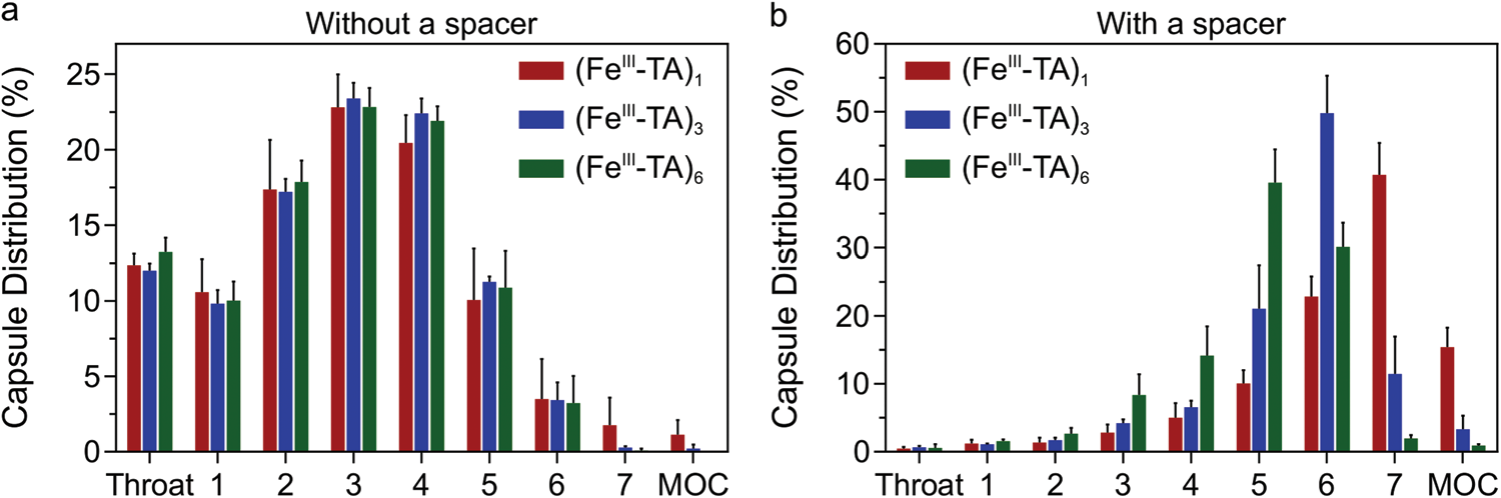
Aerodynamic behavior of the (Fe^III^‐TA)_1_, (Fe^III^‐TA)_3_, and (Fe^III^‐TA)_6_ capsules using a next generation impactor (NGI) as a lung model. Distribution of the capsules deposited in different stages of the NGI a) without or b) with a spacer connected between the nebulizer and the NGI. The NGI was precooled at 5 °C before the experiment and the air flow rate was 15 L min^−1^. The temperature and relative humidity of ambient air were 25 °C and 32–40%, respectively. The data are presented as the mean ± SD (*n* = 3).

Following nebulization, the aerodynamic behavior of the capsules was investigated using the next generation impactor (NGI) (Figure S13a, Supporting Information), which is a tool designed specifically for pharmaceutical inhaler assessment. The (Fe^III^‐TA)_1_, (Fe^III^‐TA)_3_, and (Fe^III^‐TA)_6_ capsules were labeled with thulium (Tm) via Michael addition, in which the thiol‐terminated 1,4,7,10‐tetraazacyclododecane‐1,4,7,10‐tetraacetic acid (DOTA)‐Tm complex reacts with the phenolic groups of TA under alkaline conditions.^31^ The conjugated DOTA‐Tm remained stable on the capsules after incubation for 96 h in water (Figure S14, Supporting Information), which allowed the distribution of the capsules in the NGI to be monitored. The Tm‐labeled (Fe^III^‐TA)_1_, (Fe^III^‐TA)_3_, and (Fe^III^‐TA)_6_ capsule suspensions (1 × 10^5^ capsules µL^−1^, 5 mL) were aerosolized using the air jet nebulizer and subsequently drawn into the inlet of the NGI at a flow rate of 15 L min^−1^, which represents the average inspiratory flow rate for a healthy adult human.^32^ The NGI, which models the human lung, comprises seven stages with effective cutoff diameters decreasing from 14.1 µm (representing the upper region of the respiratory tract) to 0.98 µm (representing the lower region of the respiratory tract) and a micro‐orifice collector (MOC), which acts as a final filter (Figure S13b, Supporting Information). Before the experiment, the NGI was precooled at 5 °C (close to 100% relative humidity) to minimize evaporation inside the NGI.^33^ The capsules that deposited at the various stages of the NGI were dissolved with nitric acid, and the signal from Tm in the recovered solution was measured using ICP‐MS to determine the distribution of the capsules. After 20 min of nebulization into the NGI, droplets were observed in the collection cups (Figure S15a, Supporting Information). No significant differences in NGI distribution were observed among the three capsule systems (**Figure** 4a), resulting in similar mass median aerodynamic diameters (MMADs) of ≈6.2 µm (Table S2, Supporting Information). The MMADs determined from NGI are consistent with the median size of aerosols (≈6 µm), as measured by laser diffraction (Figure S16, Supporting Information). As the mass of a single capsule (0.4–2.2 pg, Table S1, Supporting Information) is much smaller than the mass of a 6 µm water aerosol (≈113 pg), capsule deposition was mainly determined by the aerodynamic diameters of the liquid aerosol, which is influenced primarily by the nebulizer properties.

To enable control of capsule deposition, a spacer was added between the nebulizer and the NGI (Figure S17, see section Aerodynamic Behavior Analysis, Supporting Information). A spacer is commonly used in practice together with inhalation devices to produce fine aerosols by slowing down the aerosol cloud and promoting evaporation and filtration of larger aerosol particles.^34^ With the spacer in place, only the finer droplets entered the NGI, which appeared dry on the collection cups after 20 min of nebulization (Figure S15b, Supporting Information). Under these conditions, the majority (>70%) of the capsules deposited in the downstream stages (i.e., stages 5–7) of the NGI (Figure 4b), corresponding to deposition in the lower respiratory tract of the human lung—the ideal site for effective systemic delivery and for local targeting given that most respiratory diseases are located in this region.^16^ Moreover, the peak of the capsule distribution profile shifted toward the larger orifices, that is from stage 7 to stages 6 and 5 (representing a shift from the alveoli region to the terminal and secondary bronchi of a human lung),^35^ as the number of coating cycles increased (Figure 4b). Based on the NGI distribution data, the MMADs were determined as 1.31 ± 0.07, 1.85 ± 0.10, and 2.54 ± 0.20 µm for the (Fe^III^‐TA)_1_, (Fe^III^‐TA)_3_, and (Fe^III^‐TA)_6_ capsules, respectively (Table S2, Supporting Information). The increase in MMAD with the number of coating cycles is consistent with the increase in the estimated *D*_a_ of the dry capsules (0.94, 1.45, and 1.96 µm for (Fe^III^‐TA)_1_, (Fe^III^‐TA)_3_, and (Fe^III^‐TA)_6_ capsules, respectively, Table S2, Supporting Information), which results from the increasing density and diameter of the capsules as more Fe^III^‐TA complexes deposit on the capsule shell. These observations illustrate that for the fine droplets exiting the spacer, the physical properties of the capsules play a determinant role in their aerodynamic behaviors, thus highlighting the potential for fine‐tuning the aerodynamic behavior of nebulized particles for controlled pulmonary deposition by engineering their physical properties.

### 2.5 In Vivo Lung Retention and Biodegradation Study

**Figure 5:**
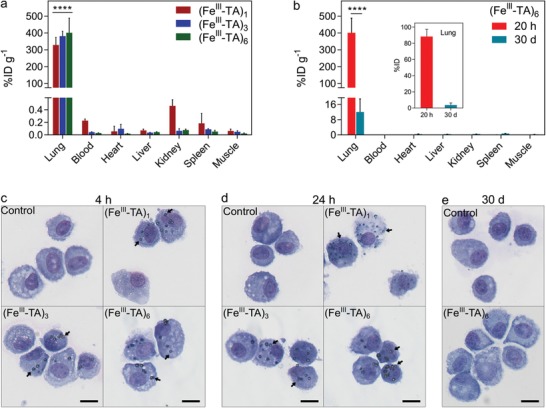
Lung retention and biodegradation study of Fe^III^‐TA capsules. a) Biodistribution of the (Fe^III^‐TA)_1_, (Fe^III^‐TA)_3_, and (Fe^III^‐TA)_6_ capsules in various organs and blood of mice at 20 h after intratracheal administration. b) Biodistribution of (Fe^III^‐TA)_6_ capsules at 20 h and 30 days after intratracheal administration. The data in (a) and (b) are presented as percentage of injected dose per gram of tissue (%ID g^−1^). The inset in (b) shows percentage injected dose (%ID) of (Fe^III^‐TA)_6_ capsules in the whole lung at 20 h and 30 days after administration. All data represent three mice per group. **** in (a) indicates *p* < 0.0001 for lung versus all other organs, whereas **** in (b) indicates *p* < 0.0001 for 20 h versus 30 days (two‐way ANOVA with Tukey's multiple comparisons test). c–e) Cytospins of bronchoalveolar lavage cells from lungs of mice at c) 4 h, d) 24 h, and e) 30 days postintratracheal administration of DPBS (control), (Fe^III^‐TA)_1_, (Fe^III^‐TA)_3_, or (Fe^III^‐TA)_6_ capsules. The arrows show examples of capsules that are associated with alveolar macrophages. Scale bars are 10 µm in (c)–(e).

Given the robustness of the capsules to nebulization and tunability for controlled pulmonary deposition, we assessed their in vivo lung retention and biodegradation. For the initial study, Tm‐labeled (Fe^III^‐TA)_1_, (Fe^III^‐TA)_3_, and (Fe^III^‐TA)_6_ capsule suspensions (1 × 10^7^ capsules in 100 µL Dulbecco's phosphate‐buffered saline (DPBS)) were intratracheally nebulized into the lung using a microsprayer under the guidance of a laryngoscope (Figure S18, Supporting Information), and the biodistribution at the organ level at 20 h postadministration was determined using ICP‐MS on digested tissue preparations. Regardless of shell thickness, the capsules showed pulmonary retention 20 h postadministration (**Figure** 5a). More than 300%ID g^−1^ of capsules remained within the lung (corresponding to >85% of the injected dose in the whole lung), whereas less than 0.5%ID g^−1^ of capsules were detected in the other organs or blood at 20 h postadministration. To examine whether these capsules were degraded and eliminated from the treated mice, the biodistribution of (Fe^III^‐TA)_6_ capsules was further studied at 30 days postadministration (Figure 5b). The (Fe^III^‐TA)_6_ capsules were chosen for the long‐term study as they contain the highest amount of shell material per capsule (Table S1, Supporting Information). Interestingly, most of the (Fe^III^‐TA)_6_ capsules were eliminated from the lung with 12%ID g^−1^ (corresponding to 3.7% of the injected dose in the whole lung) remaining after 30 days, which is 34‐fold less than that in the lung at 20 h (402%ID g^−1^). Furthermore, less than 0.5%ID g^−1^ of the capsules was detected in all the other organs and blood at 30 days, indicating the efficient elimination of the capsules from the mice. The biodegradation and inflammatory potential of the capsules introduced into the lung were further studied by examining cells in the bronchoalveolar lavage (BAL) from the lungs of treated mice (Figure 5c–e). Multiple capsules were associated with alveolar macrophages at 4 and 24 h postadministration, consistent with their uptake. However, there were no capsules observed with BAL cells 30 days postadministration, which confirms the biodistribution results obtained from ICP‐MS. Furthermore, cytospins of BAL cells from the treated mice revealed only alveolar macrophages in the lung wash and no other inflammatory cells (neutrophils, eosinophils, lymphocytes), indicating that the capsules did not induce lung inflammation (Figure 5c–e). The biodegradation of the Fe^III^‐TA capsules is likely related to the destabilization of the Fe^III^‐TA complexation at low pH,[qv: 19a] which could occur as a result of the uptake of Fe^III^‐TA capsules by alveolar macrophages into their intracellular acidic components (i.e., endosomes and lysosomes). In addition, TA displays strong interactions with mucoproteins and is biodegradable owing to the presence of hydrolyzable ester bonds connecting pyrogallol groups.^36^ Together these data indicate that the capsules are retained in the lungs for at least 20 h, can subsequently degrade and be cleared over time, and do not induce inflammation.

### 2.6 In Vivo Assessment of Lung Cell Association, Inflammation, and Toxicity of Capsules

**Figure 6:**
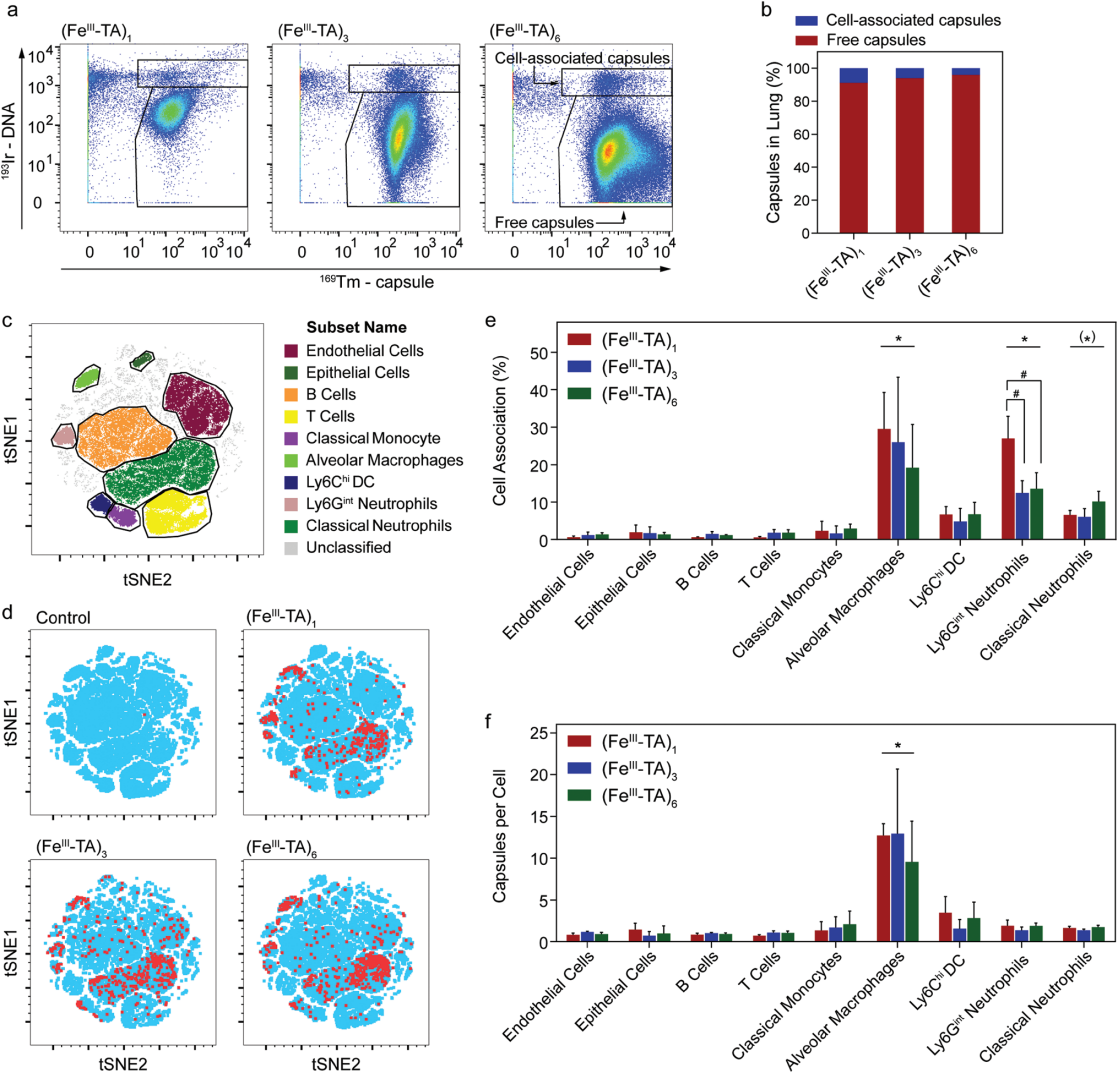
Association of capsules with lung cell populations in mice. (Fe^III^‐TA)_1_, (Fe^III^‐TA)_3_, and (Fe^III^‐TA)_6_ capsules (1 × 10^7^ capsules in 100 µL DPBS) were administered intratracheally. Following administration at 20 h, the mice were euthanized and cell suspensions were prepared from lungs for analysis by mass cytometry. a) Identification of free and cell‐associated capsules in lung digests by mass cytometry. Cell‐associated capsules were identified based on high DNA staining. b) Relative proportions of free and cell‐associated capsules in lung digests. Bars show mean values (*n* = 4). c) *t*‐Stochastic neighbor embedding (tSNE) plot from pooled lung samples showing gating of cell populations of interest. d) Representative tSNE overlay plots of mice that were administered with the indicated capsule type; red and blue dots are cells with and without capsule‐associated signals, respectively. e) Percentage of cell population showing capsule association. f) Median number of capsules per cell for the indicated cell populations. e,f) Data are shown as the mean ± SD (*n* = 4). * indicates *p* < 0.05 versus all other cell populations; (*) indicates significant versus endothelial cells, B cells, T cells, alveolar macrophages, and Ly6G^int^ neutrophils; and ^#^ indicates *p* < 0.05 between the capsule groups indicated (one‐way ANOVA with Tukey's multiple comparisons test).

Given the retention of the capsules in the lung, we assessed the relative proportion of cell‐associated and free capsules in the lung. The types of cells the capsules preferentially interacted with in the lung were determined using mass cytometry (MC), also known as cytometry by time‐of‐flight (CyToF). In this technique, cell suspensions are prepared from target organs, incubated with antibodies that have been labeled with heavy metal isotopes as cell markers, and analyzed by ICP‐MS on a single‐cell basis for antibody and capsule‐associated signal.^37^ Tm‐labeled (Fe^III^‐TA)_1_, (Fe^III^‐TA)_3_, and (Fe^III^‐TA)_6_ capsule suspensions (1 × 10^7^ capsules in 100 µL DPBS) were directly nebulized into the lungs of experimental mice, whereas control mice received DPBS only. The animals were euthanized 20 h later, and suspensions of single cells and capsules from enzymatically digested whole lungs were analyzed by MC.[qv: 37a] At 20 h post‐administration, ≈90–95% of capsules were found to be free within the lung, whereas ≈5–10% of capsules were associated with nucleated cells (**Figure** 6a,b). As a preliminary unbiased step in determining which specific cell populations the capsules associated with, a *t*‐stochastic neighbor embedding (tSNE) map was generated using the combined data from all experimental animals (Figure 6c),^38^ and the cell‐associated capsule Tm signal was overlaid (Figure 6d and Figure S19a, Supporting Information). Relevant cell populations were identified based on surface marker expression (Figure 6c, Figure S19b,c, Supporting Information).^39^ Examination of these overlays and comparison with identified populations suggested that capsules preferentially associated only with selected cell types, namely alveolar macrophages (AMs) and neutrophils (Figure 6d). Quantitative analysis confirmed that, in comparison to a range of other lung‐resident cell types, including various immune cell subsets, endothelial cells, and lung epithelial cells, the Fe^III^‐TA capsules significantly associated with AMs (20–30%) and neutrophil subsets (Ly6G^int^) (13–27%), as shown in Figure 6e. When the absolute number of capsules interacting with cells of each population was considered (Figure 6f), more capsules associated with AMs on a per cell basis (10–13 capsules per cells on average) than with any other population (a maximum of four capsules per cell). Taken together, these data are broadly consistent with the known physiology of the lung and resident cells. Alveolar macrophages are highly phagocytic cells that reside within the alveolar space and are responsible for removing particulates that penetrate to the lower levels of the respiratory tree.^40^ Targeting macrophages by their physiological ability to uptake particles is currently being proposed as a therapy for a number of diseases where macrophages are implicated, including chronic inflammation, metabolic diseases, fibrotic diseases, and cancer.^41^ Furthermore, this approach is being considered for viral infection, where macrophages are viral reservoirs, such as in acquired immune deficiency syndrome, or for chronic bacterial infection for instance in tuberculosis, where alveolar macrophages are reservoirs of the pathogenic *Mycobacterium tuberculosis*.^42^ In addition, the large proportion of capsules evading uptake by phagocytes in the lung at 20 h provides opportunities for targeting other cell populations for local drug delivery.

**Figure 7:**
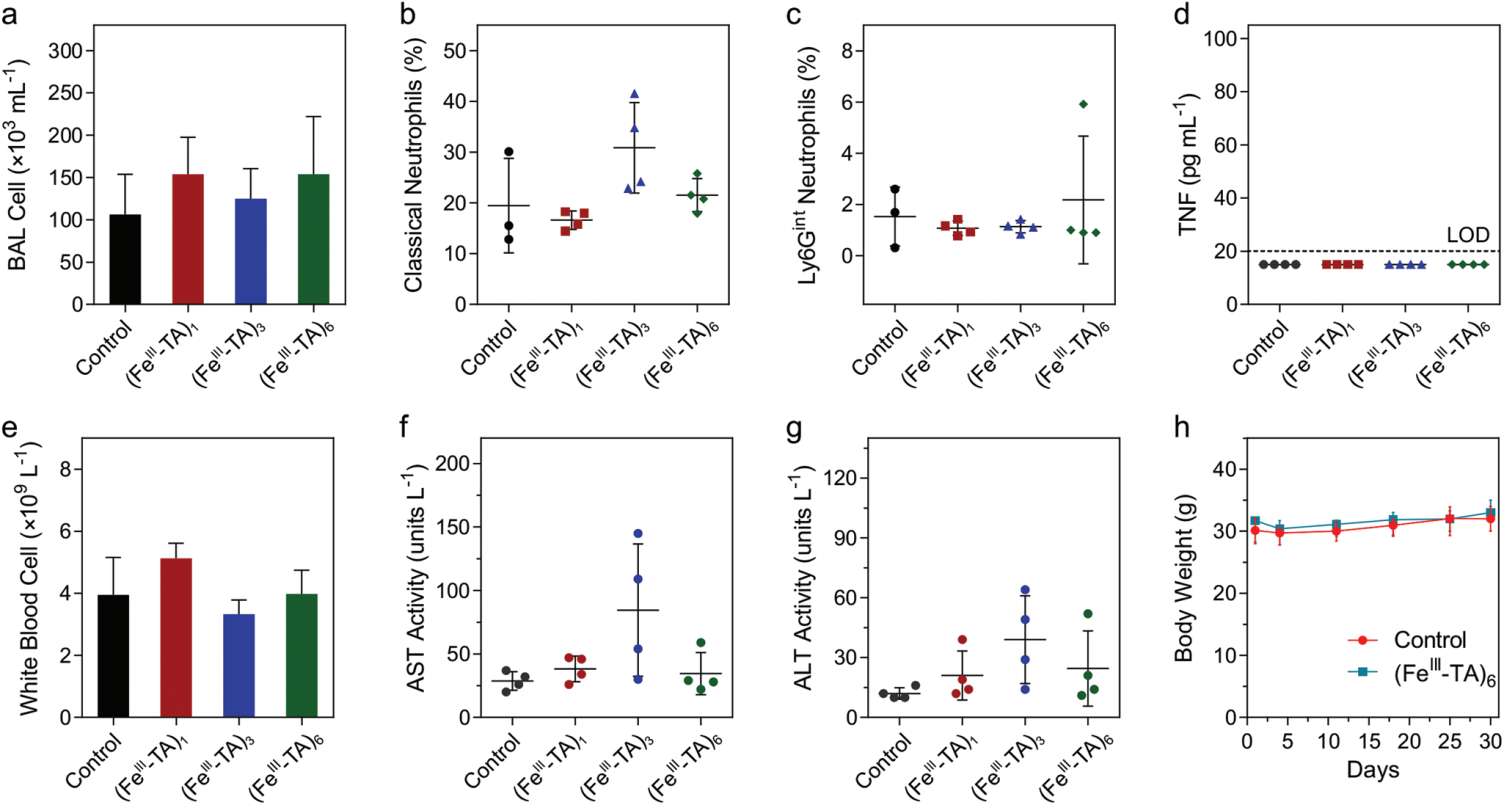
In vivo assessment of inflammation and toxicity of the capsules. Mice were administered intratracheally with either DPBS (control), (Fe^III^‐TA)_1_, (Fe^III^‐TA)_3_, or (Fe^III^‐TA)_6_ capsules (1 × 10^7^ capsules in 100 µL DPBS) and samples were collected 24 h after administration. a) Total number of bronchoalveolar lavage (BAL) cells from lungs of mice. b,c) Relative percentage of both classical and Ly6G^int^ neutrophils with respect to the total cell yield in whole lungs of mice. d) Concentration of TNF in BAL fluid of mice was below the limit of detection (LOD, 20 pg mL^−1^) of the assay. e) Concentration of circulating white blood cells from cohorts of mice. f,g) Serum levels of liver enzymes: aspartate aminotransferase (AST) and alanine aminotransferase (ALT). h) Body weight of mice over 30 days after administration. The data in (a)–(h) are shown as mean ± SD (*n* = 4); no significant differences were seen between control and (Fe^III^‐TA)_1_, (Fe^III^‐TA)_3_, or (Fe^III^‐TA)_6_ groups (one‐way ANOVA with Tukey's multiple comparisons test).

**Figure 8:**
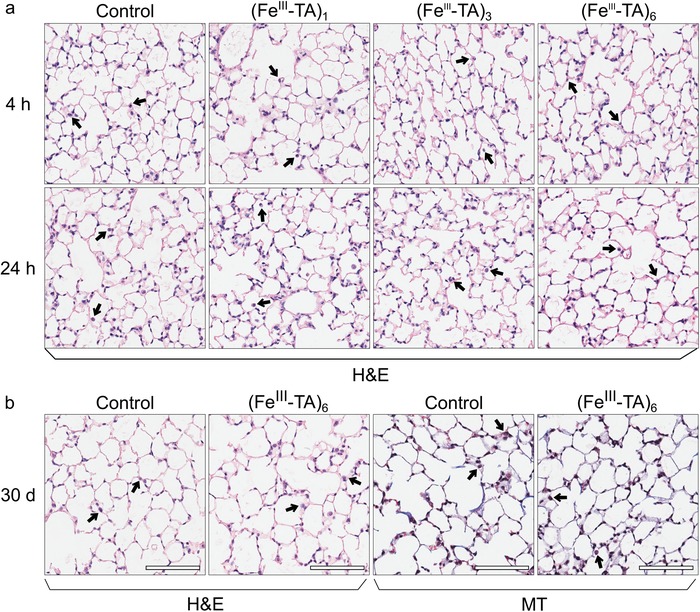
Representative histology of lung tissue at a) 4 and 24 h, and b) 30 days after intratracheal administration of DPBS (control), (Fe^III^‐TA)_1_, (Fe^III^‐TA)_3_, or (Fe^III^‐TA)_6_ capsules (1 × 10^7^ capsules in 100 µL DPBS). Alveolar architecture is maintained in all the samples. Healthy alveolar macrophages were present in all lung samples (indicated by arrows). Hematoxylin and eosin (H&E) staining for architecture and Masson's trichrome (MT) staining for fibrosis were used as indicated, 20× magnification.

Finally, the uptake of capsules by phagocytic cells could potentially cause immune activation via pattern recognition receptors, thereby leading to subsequent inflammation.^43^ Therefore, we investigated whether inflammation and subsequent lung damage occurred following pulmonary administration of the capsules. Initially, we determined the effect of capsule administration on neutrophil number because the recruitment of neutrophils to a site is a canonical feature of acute inflammation. Compared to the control mice, there was no increase in the relative proportion of the neutrophil subpopulations, with respect to the total number of cells, within the lungs of mice that were administered the capsules (Figure 5c and **Figure** 7b,c). These findings indicate that none of the capsule systems studied induce obvious inflammation in vivo. Then, a detailed investigation of the in vivo inflammatory response to the capsules, as well as in vivo toxicity, were performed. The total number of cells and levels of inflammatory cytokines in the BAL fluid (BALF), major hematological parameters, serum levels of liver enzymes and proteins, and lung histology were determined 4 and 24 h post‐administration of (Fe^III^‐TA)_1_, (Fe^III^‐TA)_3_, and (Fe^III^‐TA)_6_ capsules. A long‐term study (30 days after administration) was also conducted with (Fe^III^‐TA)_6_ capsules to reveal any potential long‐term toxicity. All mice (*n* = 4 for each system) survived the study with no signs of lethargy, ill health, change in respiration, or loss of weight after intratracheal administration of known quantities of the capsules over 30 days (Figure 7h). Compared with the control group (where mice were administered with DPBS), the administration of capsules to the lung did not result in higher BAL cell number (Figure 7a; Figure S20, Supporting Information) or higher levels of inflammatory cytokines (including MCP‐1, TNF, IFN‐γ, IL‐6, IL‐10, and IL‐12p70) (Figure 7d; Figure S21, Supporting Information). Furthermore, from lung histology, normal pulmonary architecture was observed in all experimental animals, with no signs of lung inflammation, alveolar damage, or fibrosis at 4 and 24 h and 30 days postcapsule administration (**Figure** 8). Finally, no significant changes were seen in any of the hematological parameters studied (Figure 7e; Figure S22, Supporting Information) or in serum levels of liver enzymes and proteins (Figure 7f,g; Figure S23, Supporting Information) that are indicative of toxicity. These results indicate that the Fe^III^‐TA capsules exhibit high biocompatibility and do not induce lung inflammation at the capsule dosage studied.

## 3 Conclusions

We demonstrated the synthesis of (Fe^III^‐TA)_1_, (Fe^III^‐TA)_3_, and (Fe^III^‐TA)_6_ capsules with varying shell thicknesses (≈108, ≈162, and ≈207 nm) simply by increasing the number of coating cycles (1, 3, and 6) on sacrificial CaCO_3_ templates. The increase in shell thickness had a negligible influence on cell viability and uptake by the human lung cancer cell line A549. When nebulized, the capsules did not aggregate, and the morphology of the capsules remained intact regardless of the shell thickness. The nebulization process had minimal effect on cargo leakage or release from the thicker capsules ((Fe^III^‐TA)_3_ and (Fe^III^‐TA)_6_) with less than 5% release, whereas the (Fe^III^‐TA)_1_ capsules with thinner shells released ≈31% and ≈35% of the loaded BSA and dextran, respectively. Cascade impaction studies using a mechanical lung model demonstrated that the aerodynamic behavior of the aerosolized capsules could be finely tuned by increasing the shell thickness of the capsules when a spacer was used together with the nebulizer. The aerodynamic diameter increased from 1.31 to 2.54 µm, which according to the lung model corresponds to lung deposition shifting from the alveoli to the terminal and secondary bronchi. In vivo biodistribution experiments of the Fe^III^‐TA capsules showed that >85% of the capsules remained within the lung for at least 20 h following intratracheal delivery but were degraded and cleared over time. In vivo cell association studies demonstrated that more than 90% of the Fe^III^‐TA capsules in the lung remained free (i.e., in a non‐cell‐associated form) at 20 h postadministration and the cell‐associated capsules preferentially associated with alveolar macrophages and neutrophils, which are major phagocytic cell subsets within the lung, although such association did not lead to lung inflammation or toxicity. The findings of the present study demonstrate that the Fe^III^‐TA capsules are highly attractive candidates for pulmonary delivery owing to their robustness, stability, amenability to be nebulized, potential for controlled pulmonary deposition by tuning their aerodynamic diameter, high biocompatibility, biodegradability, and their capacity for cargo loading and surface functionalization. Studies are underway to investigate the controlled release of therapeutics from Fe^III^‐TA capsules in the pulmonary environment, as well as the lung deposition in a large animal model.

## Conflict of Interest

The authors declare no conflict of interest.

## Supporting information

Supporting Information
