# Supplementary material for: Engineering of Nebulized Metal–Phenolic Capsules for Controlled Pulmonary Deposition
Source: Adv Sci (Weinh). 2020 Jan 10;7(6):1902650. doi: 10.1002/advs.201902650 (PMC7080547; doi:10.1002/advs.201902650)
Supplement: Supplementary file 1 — Supporting Information [file ADVS-7-1902650-s001.pdf]

Copyright WILEY-VCH Verlag GmbH & Co. KGaA, 69469 Weinheim, Germany, 2020.

ADVANCED SCIENCE
Open Access

Supporting Information

for *Adv. Sci.,* DOI: 10.1002/advs.201902650

Engineering of Nebulized Metal–Phenolic Capsules for Controlled Pulmonary Deposition

Yi Ju, Christina Cortez-Jugo, Jingqu Chen, Ting-Yi Wang, Andrew J. Mitchell, Evelyn Tsantikos, Nadja Bertleff-Zieschang, Yu-Wei Lin, Jiaying Song, Yizhe Cheng, Srinivas Mettu, Md. Arifur Rahim, Shuaijun Pan, Gyeongwon Yun, Margaret L. Hibbs, Leslie Y. Yeo, Christoph E. Hagemeyer, and Frank Caruso**

Copyright WILEY-VCH Verlag GmbH & Co. KGaA, 69469 Weinheim, Germany, 2018.

Supporting Information

Engineering of Nebulized Metal–Phenolic Capsules for Controlled Pulmonary Deposition

Yi Ju, Christina Cortez-Jugo, Jingqu Chen, Ting-Yi Wang, Andrew J. Mitchell, Evelyn Tsantikos, Nadja Bertleff-Zieschang, Yu-Wei Lin, Jiaying Song, Yizhe Cheng, Srinivas Mettu, Md. Arifur Rahim, Shuaijun Pan, Gyeongwon Yun, Margaret L. Hibbs, Leslie Y. Yeo, Christoph E. Hagemeyer, and Frank Caruso**

Dr. Y. Ju, Dr. C. Cortez-Jugo, J. Chen, Dr. N. Bertleff-Zieschang, J. Song, Y. Cheng, Dr. M. A. Rahim, Dr. S. Pan, Dr. G. Yun, Prof. F. Caruso
ARC Centre of Excellence in Convergent Bio-Nano Science and Technology, and the Department of Chemical Engineering, The University of Melbourne, Parkville, Victoria 3010, Australia
E-mail: fcaruso@unimelb.edu.au

Dr. T.-Y. Wang, A./Prof. C. E. Hagemeyer
Nanobiotechnology Laboratory, Australian Centre for Blood Diseases, Central Clinical School, Monash University, Melbourne, Victoria 3004, Australia
E-mail: christoph.hagemeyer@monash.edu

Dr. A. J. Mitchell
Department of Chemical Engineering, Materials Characterisation and Fabrication Platform, The University of Melbourne, Parkville, Victoria 3010, Australia

Dr. E. Tsantikos, A./Prof. M. L. Hibbs
Department of Immunology and Pathology, Central Clinical School, Monash University, Melbourne, Victoria 3004, Australia

Dr. Y.-W. Lin
Monash Biomedicine Institute, Department of Microbiology, Monash University, Clayton, Victoria 3800, Australia

Dr. S. Mettu
School of Chemistry and the Department of Chemical Engineering, The University of Melbourne, Parkville, Victoria 3010, Australia

Prof. L. Y. Yeo
Micro/Nanophysics Research Laboratory, School of Engineering, RMIT University, Melbourne, Victoria 3001, Australia

Experimental Section

Materials. $FeCl_3 \cdot 6H_2O$, Tannic acid (TA), 3-(*N*-morpholino)propanesulfonic acid (MOPS) buffer, calcium nitrate tetrahydrate ($Ca(NO_3)_2 \cdot 4H_2O$), sodium carbonate (Na_2CO_3), poly(sodium 4-styrenesulfonate) (PSS, 70 kDa), dextran–fluorescein (dextran$_{FITC}$; 500 kDa), FITC-labeled bovine serum albumin (BSA$_{FITC}$; 65 kDa), ethylenediaminetetraacetic acid (EDTA), cysteamine hydrochloride, phenazine methosulfate (PMS), triethylamine (TEA), anhydrous dimethyl sulfoxide (DMSO), Dulbecco's phosphate-buffered saline (DPBS), thulium standard for ICP, nitric acid (65%), collagenase type IV, DNAse type I, and red blood cell lysis solution were purchased from Sigma-Aldrich (USA). Iridium intercalator and Cell-ID™ cisplatin were purchased from Fluidigm (USA). Thulium(III) *S*-2-(4-isothiocyanatobenzyl)-1,4,7,10-tetraazacyclododecane-1,4,7,10-tetraacetic acid (Tm-DOTA-SCN) was purchased from Macrocyclics (USA). 2,3-Bis[2-methoxy-4-nitro-5-sulfophenyl]-2*H*-tetrazolium-5-carboxyanilide inner salt (XTT), Dulbecco's modified Eagle's medium (DMEM), and AF594-WGA were obtained from Life Technologies (USA). All chemicals were used as received without further purification. The pH of the solutions was measured with a Mettler-Toledo MP220 pH meter. Milli-Q water with a resistivity greater than 18.2 MΩ cm was obtained from a three-stage Millipore Milli-Q plus 185 purification system (Millipore Corporation, USA). All aqueous solutions were filtered with membranes with pore diameters of 220 nm before use.

Fabrication of $CaCO_3$ Templates. $CaCO_3$ particles with an average diameter of 1.1 ± 0.3 μm were prepared by a fast precipitation method in the presence of PSS, followed by calcination. To prepare PSS-stabilized $CaCO_3$ particles, 5 mL of $Ca(NO_3)_2 \cdot 4H_2O$ solution (1 M), 20 mL of PSS solution (70 kDa, 10 mg mL^{-1}), and 175 mL Milli-Q water were mixed in a 200 mL beaker under mild stirring. Next, 1 mL of Na_2CO_3 solution (1 M), 0.5 mL of PSS solution (70 kDa, 10

mg mL^{-1}), and 3.5 mL of Milli-Q water were mixed in a 50 mL tube. Subsequently, the two solutions were rapidly mixed in a 200 mL beaker under vigorous stirring for 1 min. After static incubation for 20 min, the mixed solution was again vigorously stirred for 1 min. The particles that were obtained were washed three times with Milli-Q water by centrifugation (2000 *g*, 2 min), dried in an oven, and calcined in air at 450 °C for 6.5 h.

Assembly of (Fe^{III}-TA)$_1$, (Fe^{III}-TA)$_3$, and (Fe^{III}-TA)$_6$ Capsules. To assemble the (Fe^{III}-TA)$_1$ capsules, 100 µL of $FeCl_3{\cdot}6H_2O$ solution (2 mg mL^{-1}) was added to 100 µL of $CaCO_3$ suspension (10 mg mL^{-1}), followed by vigorous stirring. Subsequently, 200 µL of TA solution (8 mg mL^{-1}) and 500 µL of MOPS buffer (20 mM, pH 8.5) were successively added to the above suspension, followed by vigorous stirring for 5 min. The particles that were obtained were washed six times with Milli-Q water by centrifugation (1000 *g*, 1 min) to remove excess material. To assemble the (Fe^{III}-TA)$_3$ and (Fe^{III}-TA)$_6$ capsules, the above coating process was repeated another two and five times, respectively. To remove the $CaCO_3$ template, the Fe^{III}-TA-coated core–shell particles were incubated with EDTA solution (100 mM, pH 7.5) for 1 min and then washed with Milli-Q water three times by centrifugation (1000 *g*, 3.5 min).

Preparation of Dextran$_{FITC}$- and BSA$_{FITC}$-Loaded (Fe^{III}-TA)$_1$, (Fe^{III}-TA)$_3$, and (Fe^{III}-TA)$_6$ Capsules. Dextran$_{FITC}$ or BSA$_{FITC}$ solution (5 mg mL^{-1}) was incubated with $CaCO_3$ particles for 3 h to allow dextran$_{FITC}$ or BSA$_{FITC}$ to infiltrate the $CaCO_3$ particles. After incubation, the particles were washed three times with Milli-Q water and directly used as templates to assemble the (Fe^{III}-TA)$_1$, (Fe^{III}-TA)$_3$, and (Fe^{III}-TA)$_6$ capsules using the same protocol described above.

Preparation of Bortezomib (BTZ)-Loaded (Fe^{III}-TA)$_1$, (Fe^{III}-TA)$_3$, and (Fe^{III}-TA)$_6$ Capsules. BTZ was loaded onto the capsules through post-modification. (Fe^{III}-TA)$_1$, (Fe^{III}-TA)$_3$, and (Fe^{III}-TA)$_6$ capsules were prepared as described above and suspended in MOPS

buffer (400 µL, 100 mM, pH 8.5). Subsequently, 50 µL of DMSO and 50 µL of BTZ (60 mM in DMSO) were successively added to the capsule suspension, followed by mild stirring. After incubation for 5 min, the capsules were washed with MOPS buffer (50 mM, pH 8.5) three times by centrifugation (1000 *g*, 3.5 min) to remove excess BTZ. To determine the BTZ loading amount, the boron signal of the capsule suspension was measured by ICP-MS (NexION-2000, PerkinElmer, USA). When measuring the BTZ loading amount after nebulization, the collected capsule suspension was washed with MOPS buffer (50 mM, pH 8.5) twice by centrifugation (1000 *g*, 3.5 min) to remove free BTZ from suspension before the ICP measurement. To determine the BTZ release profile, BTZ-conjugated $(Fe^{III}\text{-TA})_1$ capsules were incubated in 10 mM phosphate buffer at pH 6.6 over 20 h at 37 °C. The capsule suspension was kept in a Slide-A-Lyzer MINI Dialysis device (3.5k molecular weight cutoff membrane, Thermo Scientific, USA). At designated time points, 200 µL of dialyzed solution was collected for BTZ quantification by ICP-MS and replaced with the same amount of buffer (added to capsule suspension).

Preparation of Tm-Labeled $(Fe^{III}\text{-TA})_1$, $(Fe^{III}\text{-TA})_3$, and $(Fe^{III}\text{-TA})_6$ Capsules. The $(Fe^{III}\text{-TA})_n$ capsules were labeled with Tm by conjugating thiol-terminated Tm to the phenolic groups of the capsules via Michael addition. Briefly, 2 µL of Tm-DOTA-SCN (100 mg mL^{-1} in DMSO) was mixed with 21.8 µL of cysteamine hydrochloride (1 mg mL^{-1} in DMSO) at a Tm-DOTA-SCN-to-cysteamine hydrochloride molar ratio of 1:0.8. Subsequently, 0.86 µL of TEA (diluted 20× in anhydrous DMSO) was added and incubated for 2 h at 25 °C. Then, 400 µL of the $(Fe^{III}\text{-TA})_1$, $(Fe^{III}\text{-TA})_3$, or $(Fe^{III}\text{-TA})_6$-coated $CaCO_3$ particle suspension (1.5×10^6 particle μL^{-1}) in MOPS buffer (20 mM, pH 7.8) was added to the above solution and incubated for 3 h at 25 °C. After incubation, the particles were washed three times with Milli-Q water and the core was removed by EDTA, as described above.

Capsule Characterization. Differential interference contrast (DIC) and fluorescence microscopy analyses were performed on an inverted Olympus IX71 microscope (Japan). Transmission electron microscopy (TEM) and energy-dispersive X-ray spectroscopy (EDX) mapping analysis was performed on an FEI Tecnai TF20 instrument (USA) at an operation voltage of 200 kV. Scanning electron microscopy (SEM) analysis was conducted on a Philips XL30 field-emission scanning electron microscope (Netherlands) operating at a voltage of 2.0 kV. Capsule suspensions were dropped and air-dried on formvar carbon-coated gold grids and Piranha-cleaned silicon wafers for the TEM and SEM measurements, respectively. *Caution: Piranha solution is strongly oxidizing and corrosive! Extreme care should be taken during preparation and use!* The SEM samples were gold-sputtered before measurement. The concentration and fluorescence intensity of the capsules were determined using an Apogee A50-Micro flow cytometer (Apogee Flow Systems, UK). ζ-Potential of the capsules was measured by microelectrophoresis using a Zetasizer Nano-ZS (Malvern Instruments, UK). The capsules were dispersed in phosphate buffer (2 mM, pH 7.4) for ζ-potential measurement. The UV−visible absorption spectra of the capsule suspensions were measured using a Varian Cary 4000 UV−vis spectrophotometer (Varian, USA). To quantify the weight of capsules, known number of $(Fe^{III}\text{-}TA)_1$, $(Fe^{III}\text{-}TA)_3$, and $(Fe^{III}\text{-}TA)_6$ capsules, counted using an Apogee A50-Micro flow cytometer, were freeze-dried and weighed.

X-ray photoelectron spectroscopy (XPS) analysis was performed using a Kratos Axis ULTRA X-ray photoelectron spectrometer incorporating a 165 mm hemispherical electron energy analyzer. The incident radiation was monochromatic Al Kα X-rays (1486.6 eV) at 150 W (15 kV, 15 mA). Survey (wide) scans were taken at an analyzer pass energy of 160 eV and multiplex (narrow) high-resolution scans were recorded at 20 eV. Survey scans were performed in the binding energy range of 1200–0 eV with steps of 1.0 eV and a dwell time of 100 ms. Narrow high-resolution scans were run with 0.05 eV steps and 250 ms dwell time. Base

pressure was 1.0 × 10^{-9} torr in the analysis chamber and 1.0 × 10^{-8} torr during sample analysis. The spectra were processed using CasaXPS software. The survey scans were calibrated using C 1s at a binding energy of 285.0 eV and the high-resolution scans were calibrated using C–C component at a binding energy of 285.0 eV.

Mechanical Tests. The Fe^{III}-TA capsules were immobilized on a polyethylenimine (PEI)-coated FluoroDish (a cell culture petri dish with a glass base of 170 μm in thickness, World Precision Instruments Inc.). A concentrated capsule suspension (10 μL) was added to the petri dish filled with water, and the capsules were then allowed to sink and immobilize onto the PEI-coated glass base of the petri dish for 30 min. A typical optical microscopy image (100× oil immersion objective) of the immobilized capsules and the colloidal probe is shown in Figure S5. The measurements were performed on a NanoWizard II instrument (JPK) in Milli-Q water using colloidal probe cantilevers. In particular, modified cantilevers fabricated from tipless cantilevers (MLCT-O, Bruker AFM Probes) were used. Briefly, a spherical glass bead (D = 28.2 μm, Polysciences, Inc.) was glued to the cantilever using an epoxy resin (Selleys Araldite Super Strength, Selleys) through micromanipulation in an atomic force microscopy (AFM) system comprising bottom-up view optics. The glued glass bead was dried overnight for complete curing of the glue. The cantilevers were cleaned using isopropanol, water, and plasma treatment to remove any organic contamination. The spring constant of the probes was determined to be 0.122 N m^{-1} using the Hutter–Bechhoefer method.[S1] The AFM head was slowly lowered into the petri dish containing the capsules to ensure that no air bubbles were attached to the probe. The sensitivity of the probe was measured by obtaining a force curve between the bare glass base of the petri dish and the probe. A constant compliance region was used to calibrate the optical lever sensitivity, which was 23 nm V^{-1}. An example of the resulting force curve on the rigid glass substrate is shown in Figure 1e (open black circles). The location of the individual capsules was identified, and the colloidal probe was slowly lowered onto a

capsule. The vertical alignment of the probe with the capsule was achieved using the in-built micromanipulation stage. The AFM probe was then engaged automatically until a finite deflection of the probe was recorded. The capsule was then indented with the colloidal probe at a specified applied force (2 nN) and a constant piezo velocity (1 µm s^{-1}), and the resulting force–distance curves were recorded. Force–distance curves of 15 different capsules per system were collected and analyzed using JPK data processing software.

Reissner's theory developed in 1947 has been widely used to estimate the stiffness of capsules.[S2] A number of modifications have since been proposed by Berry *et al*. to account for the correct indentation of the capsules.[S3] The modified theory uses the effective indentation of the capsule at the top ($\delta_{\mathrm{eff}} = C\delta_{\mathrm{AFM}}$) instead of the indentation measured by AFM (δ_{AFM}). Additionally, Berry *et al*. numerically calculated the effective indentation as a function of the radius of the AFM probe (R_{p}), the radius of the capsule (R), and the shell thickness (h). The modified theory, which has been used to calculate the stiffness values in this study, is described as follows:

$$F = \frac{4h^3}{R\sqrt{3(1-v^2)}} E_s \beta \left(C \frac{\delta_{\mathrm{AFM}}}{h} \right)^{\alpha} \quad \text{(S1)}$$

where F is the measured force as a function of indentation of probe into the capsules (δ_{AFM}), E_{s} is the Young's modulus of the shell, v is the Poisson ratio, β, C, and α are the correction factors that depend on shell thickness, probe-to-capsule radius ratio, and Poisson ratio, respectively, as tabulated by Berry *et al*.[S3] We have used a Poisson ratio of 0.4 as the correction factors are available for this value.

Cell Culture. Human alveolar adenocarcinoma epithelial A549 cells (American Type Culture Collection, USA) were cultured in complete DMEM media containing 10% fetal bovine serum

(FBS) and 2 mM L-glutamine at 37 °C with 5% CO_2 and 95% relative humidity. A549 cells with passage number 20–30 were used in the study and all cells passed the mycoplasma test.

Cell Association Analysis by Flow Cytometry. A549 cells were seeded on a 24-well plate at a cell density of 8 × 10^4 cells per well and allowed to adhere overnight at 37 °C with 5% CO_2. Subsequently, $dextran_{FITC}$-loaded $(Fe^{III}\text{-TA})_1$, $(Fe^{III}\text{-TA})_3$, and $(Fe^{III}\text{-TA})_6$ capsules were added at a cell-to-capsule ratio of 1:100 and incubated for predetermined time intervals at 37 °C with 5% CO_2. After incubation, the cells were gently washed three times with DPBS, harvested by trypsinization, and analyzed by an Apogee A50-Micro flow cytometer (Apogee Flow Systems, UK). The experiments were conducted in triplicate with at least 8000 cells analyzed in each experiment. Cells that displayed stronger fluorescence intensity than untreated cells were identified as those that associated with the capsules. The gating strategy used for the flow cytometry analyses is shown in Figure S24.

Cell Internalization Analysis by Imaging Flow Cytometry. A549 cells were seeded on a 6-well plate at a cell density of 5 × 10^5 cells per well and incubated overnight. Then, $dextran_{FITC}$-loaded $(Fe^{III}\text{-TA})_1$, $(Fe^{III}\text{-TA})_3$, and $(Fe^{III}\text{-TA})_6$ capsules were incubated with the cells at a cell-to-capsule ratio of 1:50 (a lower capsule-to-cell ratio was used for imaging flow analysis to reduce background noise) for 6 h at 37 °C. Following incubation, the cells were washed three times with DPBS, trypsinized, and collected by centrifugation (300 *g*, 5 min). The collected cells were fixed with paraformaldehyde (4% in DPBS) overnight at 4 °C and then stained with AF594-WGA (5 µg mL^{-1}) for 5 min at 4 °C, followed by washing with DPBS three times by centrifugation (1000 *g*, 10 min). Finally, the cell pellets were resuspended in DPBS (50 µL) and kept on ice until the analysis was conducted using imaging flow cytometry (AMNIS ImageStream®^{X} MarkII, Amnis Corporation, USA). Bright-field and fluorescence images of at least 5000 cells were acquired. The internalization analysis was performed using a built-in

internalization function of the Amnis ImageStream IDEAS software on single focused cells associated with the capsules.

Cell Viability Analysis by XTT Assay. A549 cells were seeded on a 96-well plate at a density of 5000 cells per well and incubated overnight. The cells were then incubated with the (Fe^{III}-TA)$_1$, (Fe^{III}-TA)$_3$, and (Fe^{III}-TA)$_6$ capsules at varying cell-to-capsule ratios from 1:10 to 1:200 (corresponding to 0.2–4.0 μg mL^{-1} for (Fe^{III}-TA)$_1$ capsules, 0.6–11.0 μg mL^{-1} for (Fe^{III}-TA)$_3$ capsules, and 1.1–22 μg mL^{-1} for (Fe^{III}-TA)$_6$ capsules) for 48 h. After treatment, the culture media was replaced with fresh DMEM media (100 μL) containing XTT and PMS (9 mL of 0.2 mg mL^{-1} XTT in complete DMEM media and 22.5 μL of 0.6 mg mL^{-1} PMS in DPBS), and the cells were incubated for a further 4 h. Subsequently, the absorbance at a wavelength of 475 nm was measured by an Infinite M200 microplate reader (Tecan, Switzerland). The relative cell viability (%) was determined by normalizing the absorbance of the treated cells as a percentage of the untreated cells. The experiments were performed in quadruplicate and the data are presented as the mean ± SD.

Aerosol Collection. The aerosol or mist from the air-jet nebulizer was collected by attaching a pipette tip to the outlet of the nebulizer and fixing it in place with parafilm (Figure 3a3). During nebulization, the mist deposits and accumulates on the walls of the pipette tip as a capsule suspension, which can then be collected by pipetting directly into a tube (1.5 mL Eppendorf tube) for further analysis.

Aerosol Characterization. The real-time size distribution of aerosols from the air-jet nebulizer were characterized by laser diffraction (Spraytec, Malvern, UK). The Spraytec was equipped with an inhalation cell connected to the Next Generation Impactor (NGI) to provide an air flow rate of 15 L min^{-1}. Capsule solutions were nebulized using a PARI air-jet nebulizer and the size distribution was measured at the inhalation cell inlet.

Aerodynamic Behavior Analysis. Following nebulization of the capsule suspension via a PARI air-jet nebulizer (Trek® S Portable Aerosol System, PARI, USA), the aerodynamic behavior of the capsules was evaluated using an NGI (Copley Scientific Limited, UK). The NGI was pre-cooled at 5 °C in the fridge before the experiment. The temperature and relative humidity of ambient air were 25 °C and 32–40%, respectively. The outlet of the NGI was connected to a vacuum pump that was set to draw air through the inlet of the system at 15 L min^{-1} to mimic adult tidal breathing. The nebulizer contained 5 mL of Tm-labeled $(Fe^{III}\text{-}TA)_1$, $(Fe^{III}\text{-}TA)_3$, or $(Fe^{III}\text{-}TA)_6$ capsule suspension (1×10^5 capsules µL^{-1} in Milli-Q water). After switching on the nebulizer, the aerosolized capsules were either directly drawn into the NGI or passed through a Volumatic® spacer (GlaxoSmithKline UK Ltd) before entering the NGI (Figure S13a and Figure S17). The joint of the spacer and the inlet of the NGI was sealed with parafilm. The capsule solution was nebulized for ~20 min during the experiment. All capsule samples were run in triplicate. After each measurement, the capsules in the inlet (i.e., throat) and at each stage of the NGI were dissolved with 5 mL of nitric acid (5%). The resulting solution was collected and subsequently analyzed by ICP-MS to determine the amount of Tm in each stage. Flow cytometry allows determination of the number of the capsules, and the Tm concentration in the capsule suspension was determined by ICP-MS. From these data, the amount of Tm per capsule was estimated as the total Tm concentration divided by the total number of capsules in the suspension. Subsequently, the number of capsules in each stage of the NGI could be determined based on the amount of Tm in each stage of the NGI. The capsule distribution (%) in the NGI was expressed as the percentage of capsules deposited in one stage relative to the total amount of capsules nebulized into the NGI. Based on the capsule distribution data, the mass median aerodynamic diameter (MMAD) was determined using an algorithm based on ISO 27427 (Nebulizing Systems and Components) via MMAD Calculator (http://www.mmadcalculator.com/).

In Vivo Studies. All procedures were conducted in accordance with the Australian National Health and Medical Research Council's published Code of Practice for the Use of Animals in Research, and experiments were approved by the Alfred Medical Research and Education Precinct (AMREP) Animal Ethics Committee (E/1625/2016/M). Mice were housed on a 12 h light/dark cycle with ad libitum access to food and water.

Biodistribution Study at Organ Level and Single-Cell Level. To evaluate the biodistribution of the Fe^{III}-TA capsules in vivo, male C57BL/6 mice weighing around 25–30 g were first anesthetized by isoflurane inhalation (initial dose 5% in oxygen to achieve anesthesia, reduced to 2% to maintain sedation) before intratracheal nebulization of Tm-labeled $(Fe^{III}\text{-TA})_1$, $(Fe^{III}\text{-TA})_3$, and $(Fe^{III}\text{-TA})_6$ capsule suspensions (1×10^7 capsules in 100 µL DPBS) using a Penn-Century microspray/syringe assembly (MSA-250-M, Penn-Century Inc., USA) under the guidance of a small animal laryngoscope (LS-2-M, Penn-Century Inc., USA). After administration, the mice were closely monitored for the next 3 h and at the experimental endpoint. The animals were then euthanized at defined time points. Their blood was collected by cardiac puncture, and their organs, including lung, heart, liver, kidney, spleen, and muscle, were collected and weighed after perfusion with DPBS. For the organ level biodistribution study, all the organs, including muscle and blood, were digested with 2 mL of 65% nitric acid at 70 °C for 2 h. *Caution! Extreme care should be taken when handling 65% nitric acid, which can only be used in a fume hood.* The Tm concentration in each organ was determined by ICP-MS. As the amount of Tm per capsule was predetermined by flow cytometry (capsule number count) and ICP-MS (Tm concentration in the capsule suspension), the total number of capsules in each organ was determined based on the amount of Tm in each organ. The injected dose (%) was expressed as the percentage of capsules detected in one organ relative to the total amount of capsules nebulized into the mice. The total volume of blood in a mouse accounts for ~7% of the body weight and the density of blood is 1.06 g mL^{-1}. These figures were considered when

calculating the number of capsules in blood. For the single-cell level biodistribution study, the collected lungs were stored in DPBS on ice prior to preparation for mass cytometry analysis (detailed in the following section).

Mass Cytometry Sample Preparation and Analysis. Lungs from mice were finely minced and digested in DPBS (4 mL) containing collagenase type IV (5 mg mL^{-1}) and DNASE-I (280 U mL^{-1}). Samples were incubated at 37 °C for 30 min, triturated through a Pasteur pipette to disperse clumps, and incubated further for 30 min at 37 °C, after which a second trituration step was performed. After digestion, the samples were centrifuged for 5 min at 350 *g* at 24 °C and the supernatant was removed. Red blood cells were then lysed by addition of the red blood cell lysis buffer (2 mL) for 7 min at 24 °C. After lysis, 20 mL of CyTOF buffer (CFB; DPBS containing 1% BSA, 5 mM EDTA, and 0.05% sodium azide) was added. The samples were then centrifuged for 5 min at 350 *g* at 24 °C, and the supernatant was removed. A second wash in CFB (10 mL) was performed before the cells were resuspended in CFB (1 mL), filtered through a 70 µm mesh, and used for staining. Staining was performed using a combination of pre-conjugated and in-house conjugated antibodies as outlined in Table S2. The cells were initially incubated with optimally titrated concentrations of primary antibodies for 40 min on ice and then washed as described above using CFB (1 mL). Dead cells were stained by resuspending cells in 0.5 mL of a 1/4000 dilution of cisplatin viability reagent in DPBS for 5 min at room temperature. Cisplatin staining was terminated by addition of CFB (1 mL) and cells were washed twice by centrifugation. Secondary antibody staining was then performed using a metal-labeled anti-biotin secondary conjugate for 20 min on ice, and cells were subsequently washed twice with CFB (1 mL). Fixation and nuclear staining were performed by addition of 400 µL of 4% formaldehyde containing Cell-ID^{TM} intercalator iridium (Ir) (0.1 µM, Fluidigm, USA) at 4 °C for 24 h. On the day of sample acquisition, the cell suspensions were washed twice by centrifugation in distilled water (1 mL, 900 *g*, 10 min, 24 °C) and

resuspended in water containing a 1/10 dilution of EQ-beads (Fluidigm, USA) at a concentration of ~1 × 10^{6} cells mL^{-1}. Data were acquired on a Helios mass cytometer (Fluidigm, USA), and post-acquisition signal normalization was performed using Helios software (Fluidigm, USA). The signal intensity of the capsules was determined by running suspensions, appropriately diluted in water, on the Helios mass cytometer.

Mass Cytometry Data Analysis. Data from all mice were concatenated, and gating performed as shown in Figure S19a using FlowJo v10-CL (Treestar). Dimensionality reduction was conducted on viable singlet events from the concatenated samples by tSNE[71] using surface markers (CD45, CD11b, CD19, CD3, B220, CD200R, NK1.1, MHC-II, Siglec-F, CD301, CD209b, Cx3cr1, CD335, CD90.2, CD326, Ly6C, Ly6G, CD11c, CD64, CD16/32, F4/80, and CD31) and the following tSNE parameters: iterations = 1000, perplexity = 20, and θ = 0.5. Because free capsules bound appreciable amounts of both the iridium intercalator and cisplatin and confounded initial interpretation of the data (not shown), these were excluded by Boolean gating ($^{169}Tm^{pos}$ events not expressing lineage markers) as shown in Figure S19a. Cell populations in tSNE plots were identified based on characteristic patterns of surface marker expression (endothelial cells: $CD45^{-}CD326^{-}CD31^{+}$; epithelial cells: $CD45^{-}CD326^{+}CD31^{-/lo}$; B cells: $CD326^{-}CD31^{-}CD45^{+}CD3^{-}CD19^{+}B220^{+}$MHC-$II^{+}$; T cells: $CD326^{-}CD31^{-}CD45^{+}CD19^{-}B220^{-}CD90^{+}CD3^{+}$; classical ($Ly6C^{hi}$) monocytes: $CD326^{-}CD31^{-}CD45^{+}CD3^{-}CD19^{-}B220^{-}CD11b^{+}SiglecF^{-}Ly6G^{-}$MHC-$II^{-/lo}Ly6C^{hi}$; alveolar macrophages: $CD326^{-}CD31^{-}CD45^{+}CD3^{-}CD19^{-}B220^{-}CD11b^{-/lo}Ly6G^{-}SiglecF^{+}F4/80^{+}CD64^{+}$; $Ly6C^{hi}$ dendritic cells: $CD326^{-}CD31^{-}CD45^{+}CD3^{-}CD19^{-}B220^{-}CD11b^{+}SiglecF^{-}Ly6G^{-}MHC^{-}II^{hi}Ly6C^{hi}$; $Ly6G^{int}$ neutrophils: $CD326^{-}CD31^{-}CD45^{+}CD3^{-}CD19^{-}B220^{-}CD11b^{+}SiglecF^{-}Ly6G^{int}$MHC-$II^{-}Ly6C^{int}$; classical ($Ly6G^{hi}$) neutrophils:

$CD326^{-}CD31^{-}CD45^{+}CD3^{-}CD19^{-}B220^{-}CD11b^{+}SiglecF^{-}Ly6G^{hi}MHC\text{-}II^{-}Ly6C^{int}$; Figure S19b). Population identification was confirmed using hierarchical gating followed by mapping onto tSNE plots (data not shown). Populations of interest were then gated from the tSNE plot for analysis of the ^{169}Tm signal (Figure S19c). The percentage of each population, from individual mice, that had interacted with the capsules was determined. The number of capsules associated with cell subsets for a given population and experimental animal was calculated by dividing the median ^{169}Tm signal of capsule^{pos} events by the median intensity value of capsules.

Bronchoalveolar Lavage (BAL) and Cytospins. Lungs from lethally anesthetized mice were lavaged using 400 µL DPBS. BAL cells were counted, centrifuged onto glass slides using a Shandon CytoSpin 4 cytocentrifuge (Thermo Fisher Scientific, USA), stained with Diff-Quik (Merck, Germany), and mounted. The BAL fluid (BALF) was centrifuged to pellet cells, and the supernatant was kept for cytokine measurement.

Lung Fixation and Histology. Lungs were inflation-fixed at 25 cm H_2O pressure in 10% neutral-buffered formalin and then paraffin embedded. Whole lung sections (3 µm) were stained with H&E or Masson's trichrome and scanned with an Aperio microscope and scanner (Leica Biosystems, Germany).

Cytokine Expression, Hematology, and Liver Toxicity Study. The cytokine expression in the BALF of mice was quantified using a BD cytometric bead array mouse inflammation kit (BD Biosciences, USA) according to the manufacturer's instructions. For the hematology study, fresh blood from mice was analyzed using a HEMAVET blood analysis system (Cell-DYN Emerald, USA). For liver toxicity studies, fresh blood samples from mice were assayed for serum levels of liver enzymes and proteins using the Alfred pathology service (Alfred Health, Australia).

Statistical Analysis. Statistical analysis were carried out using GraphPad software Prism (Version 6.0) by one-way ANOVA with Tukey's multiple comparisons test for Figures 2c, 6e,f, and 7a–g and Figures S20, S22, and S23 and two-way ANOVA with Tukey's multiple comparisons test for Figures 3e,f and 5a,b. In all cases, significance was defined as $p \leq 0.05$.

Minimum Information Reporting in Bio–Nano Experimental Literature (MIRIBEL). The studies conducted herein, including material characterization, biological characterization, and experimental details, conform to the MIRIBEL reporting standard for bio–nano research,[S4] and a companion checklist of these components is provided as part of the Supporting Information.

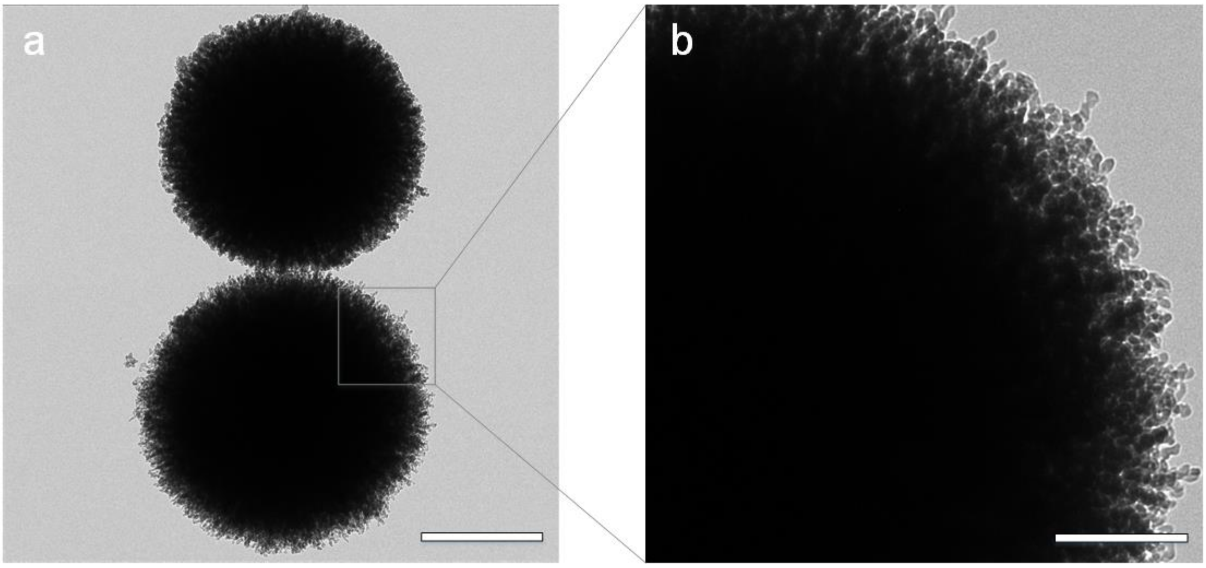

Figure S1. TEM images of $CaCO_3$ particles showing their porous surface structure. Scale bars: (a) 500 and (b) 200 nm.

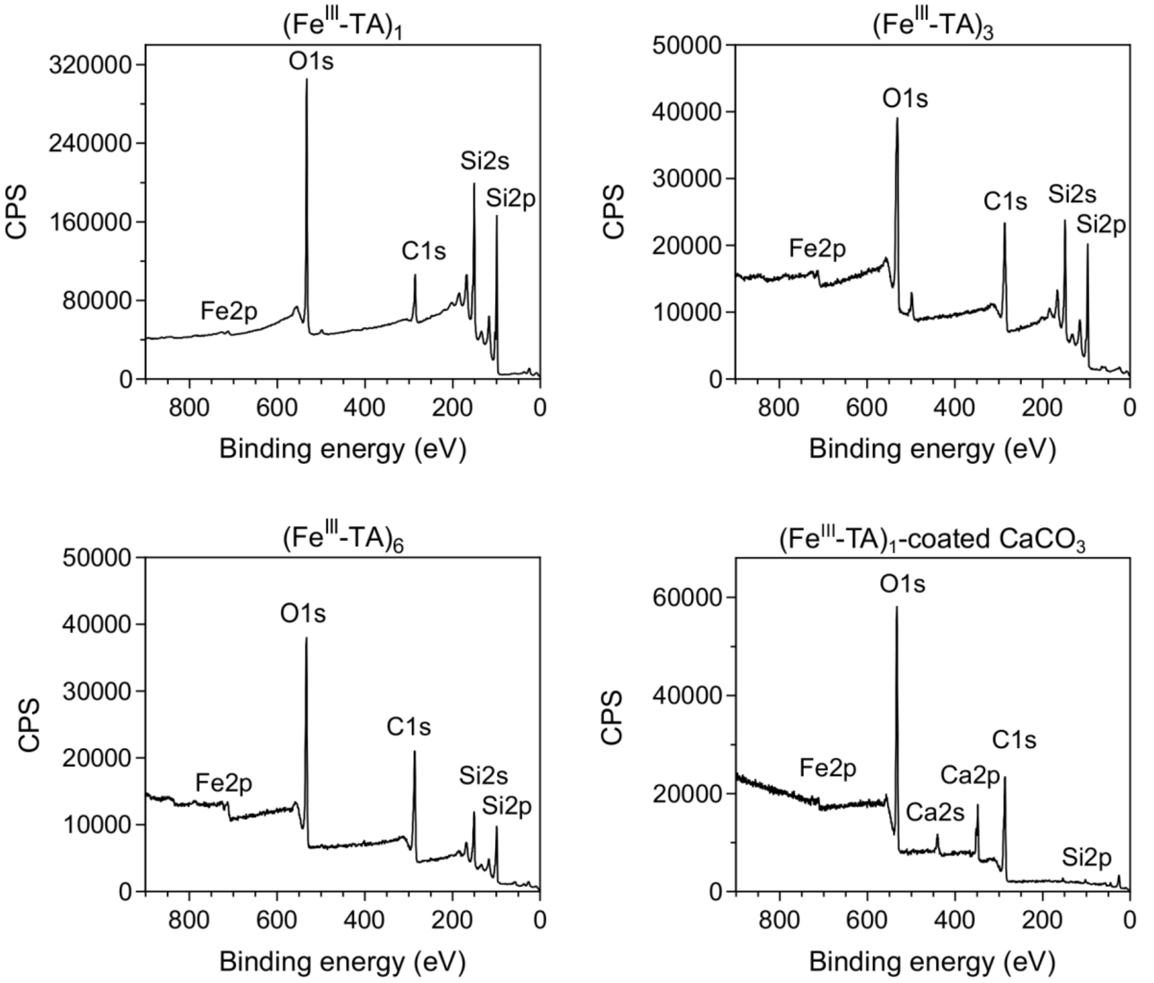

Figure S2. XPS spectra of the (Fe^{III}-TA)$_1$, (Fe^{III}-TA)$_3$, (Fe^{III}-TA)$_6$ capsules and (Fe^{III}-TA)$_1$-coated $CaCO_3$. The Si 2s and Si 2p signals are from the silicon wafer substrates. Nitrogen and calcium signals, which could arise from the residual presence of EDTA and $CaCO_3$, respectively, were not detected from either of the three capsule systems.

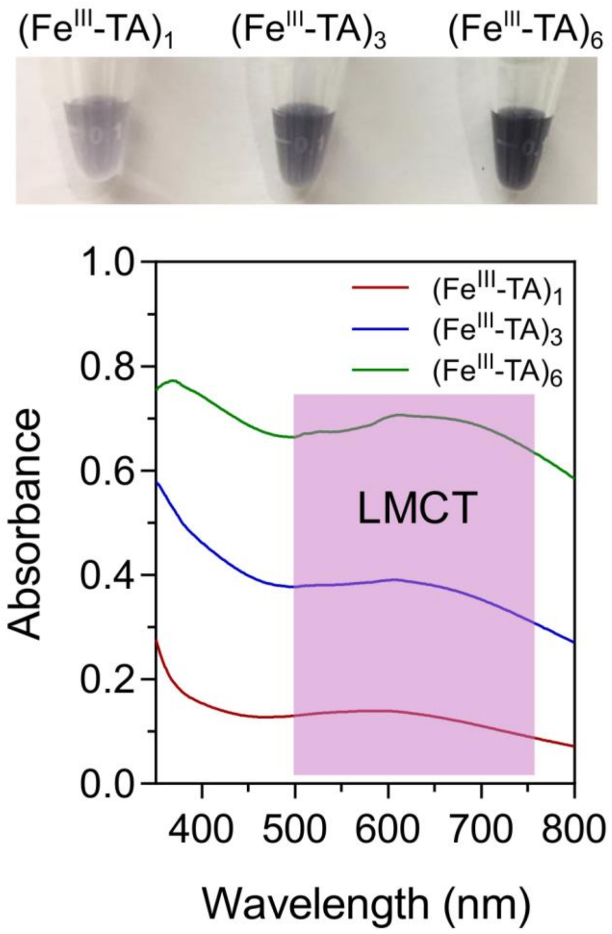

Figure S3. Photograph and UV–visible absorbance spectra of the $(Fe^{III}\text{-}TA)_1$, $(Fe^{III}\text{-}TA)_3$, and $(Fe^{III}\text{-}TA)_6$ capsule suspensions in water. The ligand-to-metal charge transfer (LMCT) band is shown in the pink region. The concentration of the capsules with one, three, or six cycles of coating was 2.7×10^7 capsules in 400 μL water.

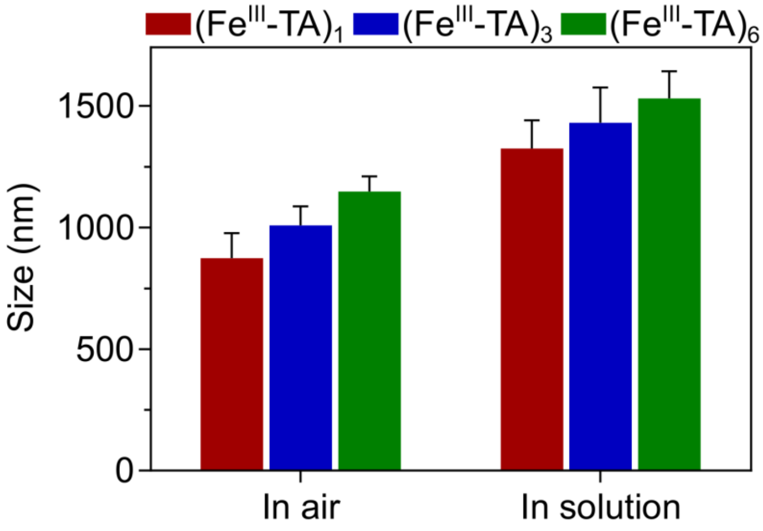

Figure S4. Diameters of the (Fe^{III}-TA)$_1$, (Fe^{III}-TA)$_3$, and (Fe^{III}-TA)$_6$ capsules when dispersed in water, as measured from DIC microscopy images, or when air-dried, as measured from TEM images (mean ± SD, n = 30).

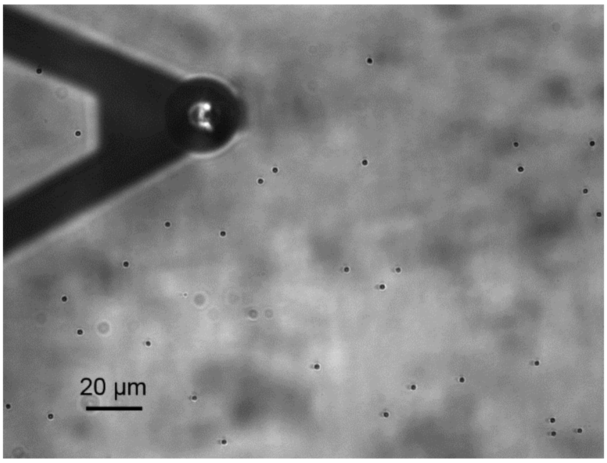

Figure S5. Optical microscopy image of the (Fe^{III}-TA)$_6$ capsules attached to the PEI-coated glass base of a petri dish and the silica colloidal probe used for the colloidal-probe atomic force microscopy measurement.

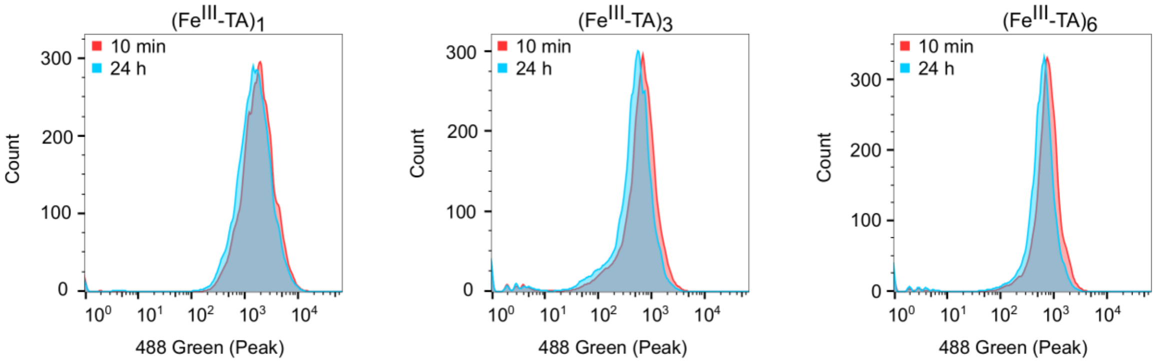

Figure S6. Overlay of flow cytometry histograms (488 Green channel, emission 500–540 nm, plotted against count) of the dextran$_{FITC}$-loaded (Fe^{III}-TA)$_1$, (Fe^{III}-TA)$_3$, and (Fe^{III}-TA)$_6$ capsules comparing the fluorescence intensity of the loaded capsules after incubation in DMEM with 10% FBS at 37 °C for 10 min and 24 h.

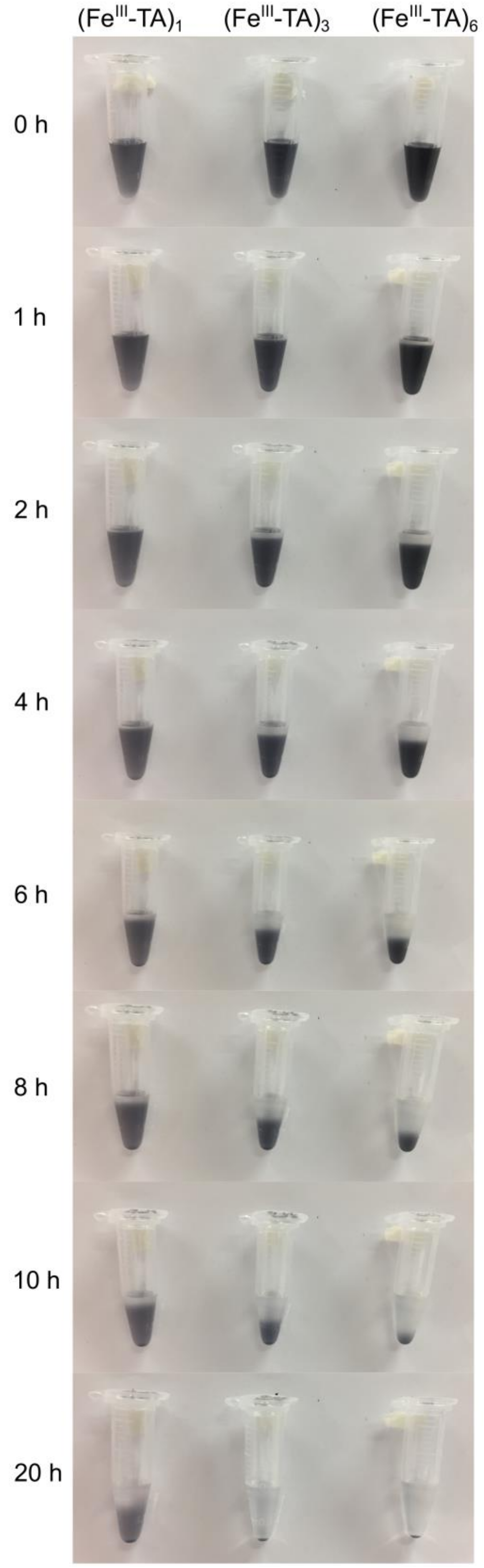

Figure S7. Photograph of the (Fe^{III}-TA)$_1$, (Fe^{III}-TA)$_3$, and (Fe^{III}-TA)$_6$ capsule suspensions in water showing the sedimentation of capsules over 20 h. The concentration of the capsules with one, three, or six cycles of coating was 1.7×10^8 capsules in 400 μL water.

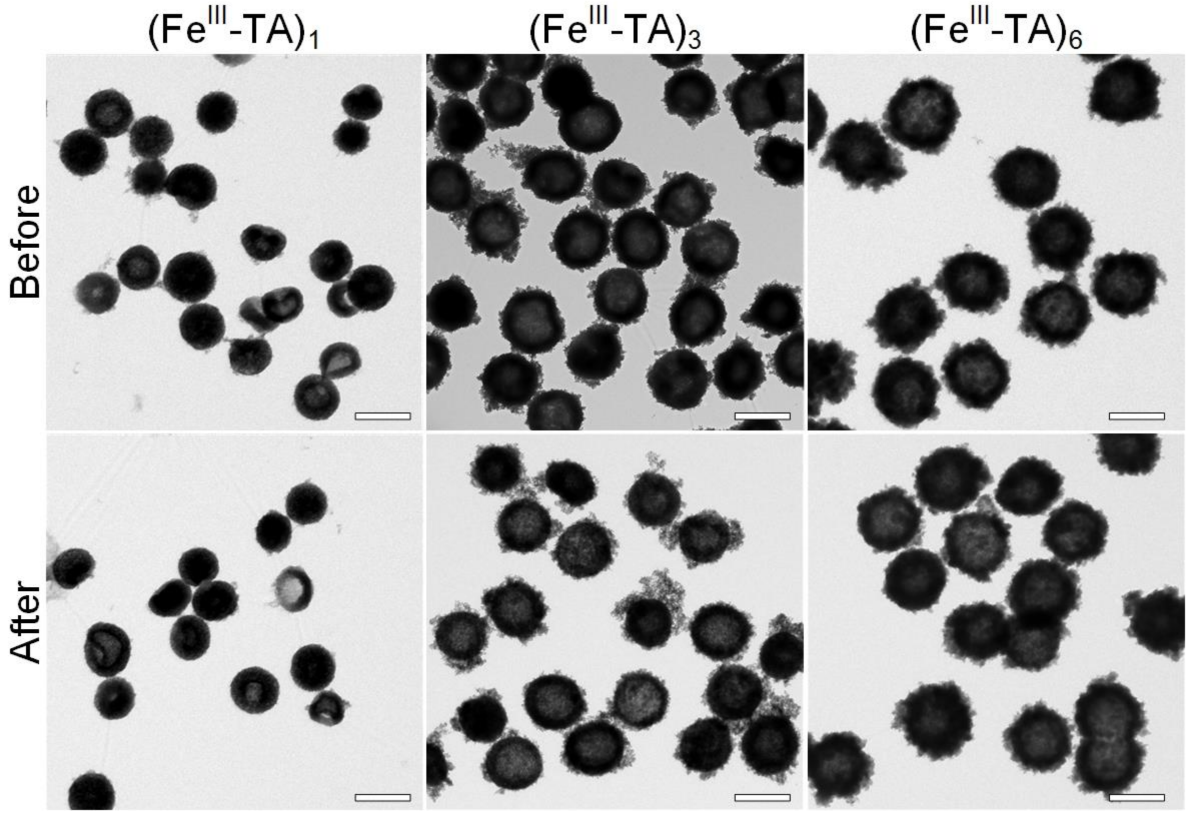

Figure S8. TEM images of the $(Fe^{III}\text{-}TA)_1$, $(Fe^{III}\text{-}TA)_3$, and $(Fe^{III}\text{-}TA)_6$ capsules before and after nebulization. Scale bars: 1 μm.

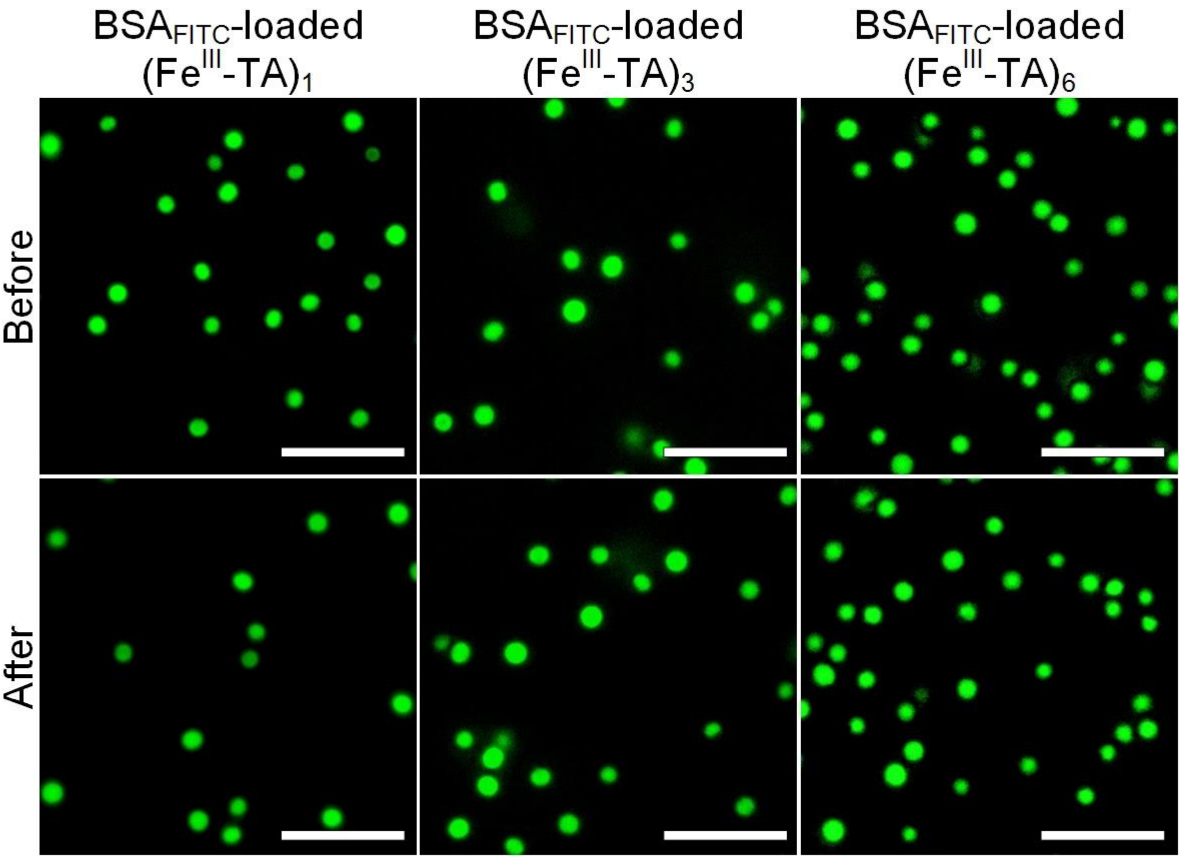

Figure S9. Fluorescence microscopy images of the BSA$_{FITC}$-loaded $(Fe^{III}\text{-}TA)_1$, $(Fe^{III}\text{-}TA)_3$, and $(Fe^{III}\text{-}TA)_6$ capsules before and after nebulization. Scale bars: 10 μm.

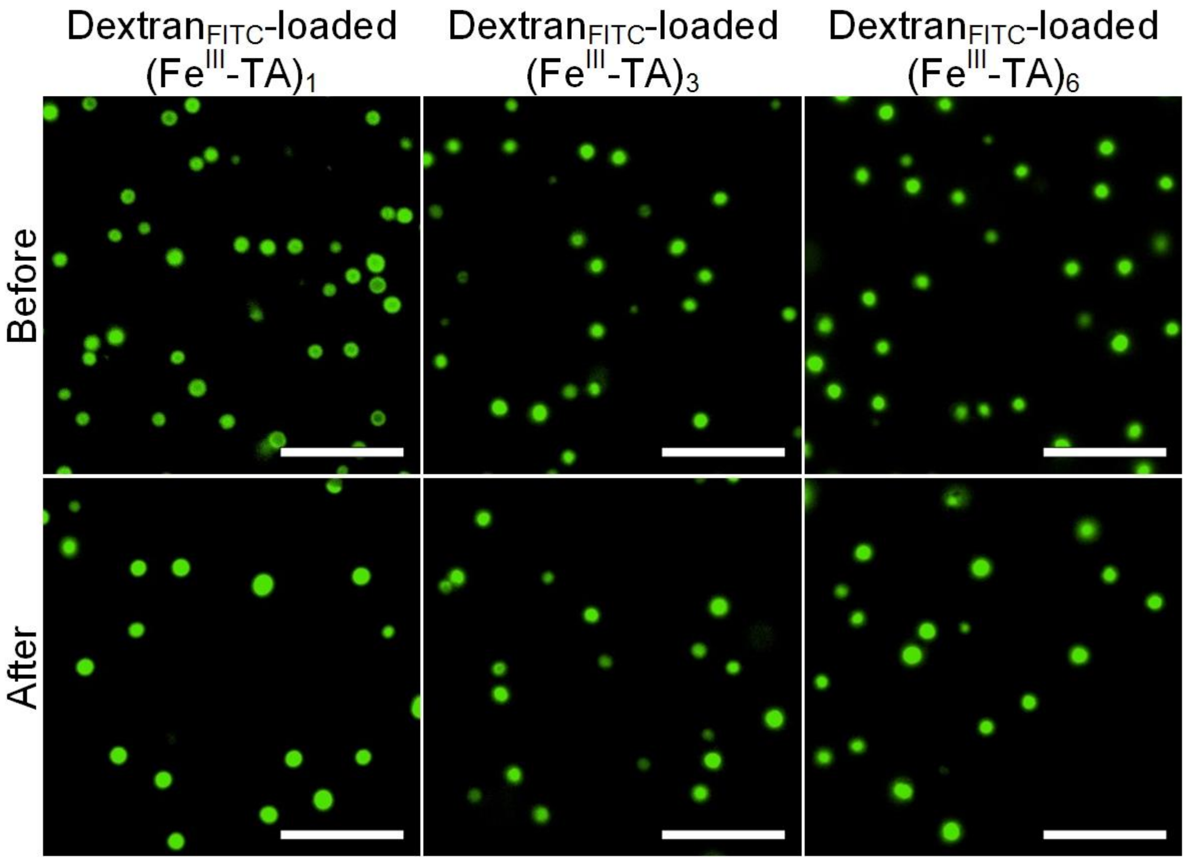

Figure S10. Fluorescence microscopy images of the dextran$_{FITC}$-loaded $(Fe^{III}\text{-TA})_1$, $(Fe^{III}\text{-TA})_3$, and $(Fe^{III}\text{-TA})_6$ capsules before and after nebulization. Scale bars: 10 μm.

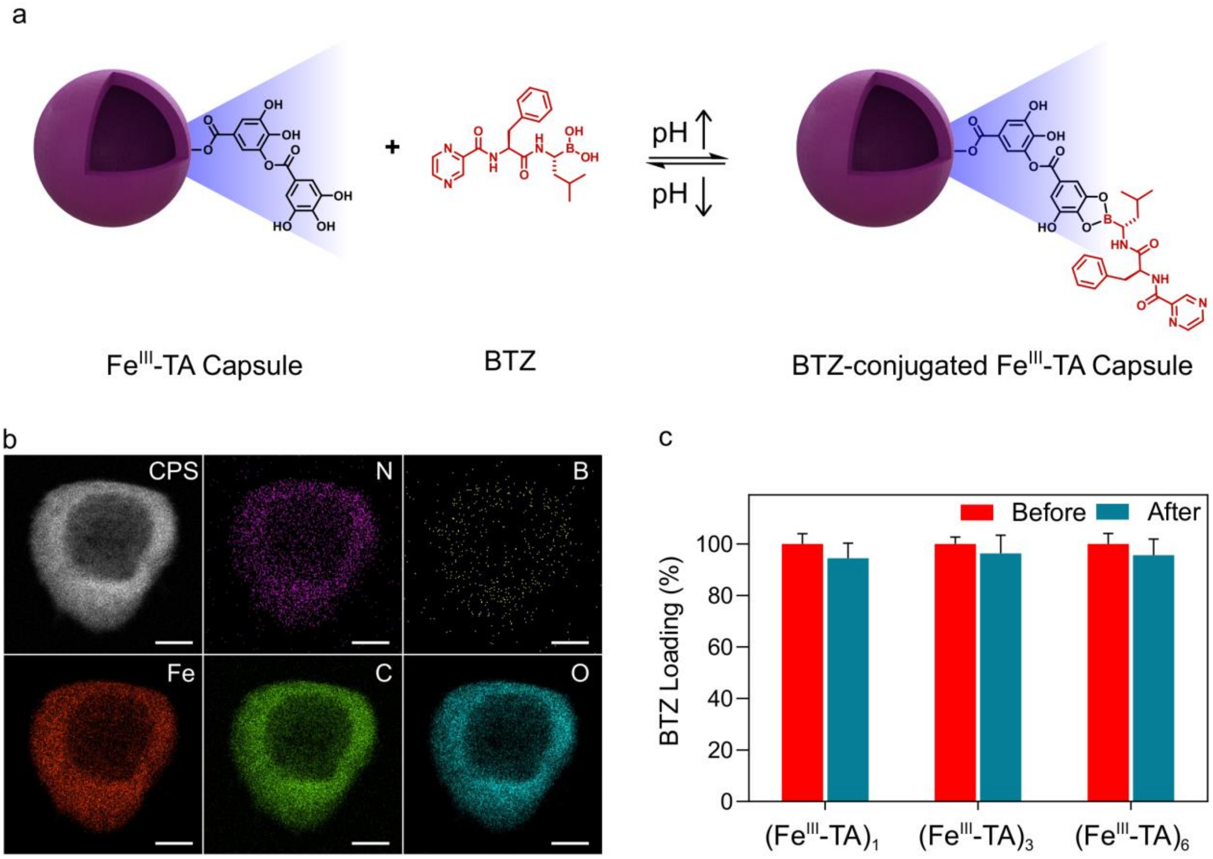

Figure S11. (a) Schematic illustration of the conjugation of bortezomib (BTZ) to Fe^{III}-TA capsules through pH-dependent boronic acid–phenol complexation. (b) EDX mapping analysis of a BTZ-conjugated (Fe^{III}-TA)$_1$ capsule. The signals of N and B (from BTZ) were observed to closely match the signals of Fe, C, and O (from the (Fe^{III}-TA)$_1$ capsule), demonstrating the successful conjugation of BTZ to the capsule. Scale bars are 0.2 µm. (c) Relative loading amount of BTZ on the (Fe^{III}-TA)$_1$, (Fe^{III}-TA)$_2$, and (Fe^{III}-TA)$_3$ capsules before and after nebulization. The loading amount of BTZ on the capsules prior to nebulization was normalized to 100% to allow comparison with the loading amount obtained following nebulization. No significant differences in BTZ loading were observed before and after nebulization for each capsule system (mean ± SD, n = 3, two-way ANOVA with Tukey's multiple comparisons test).

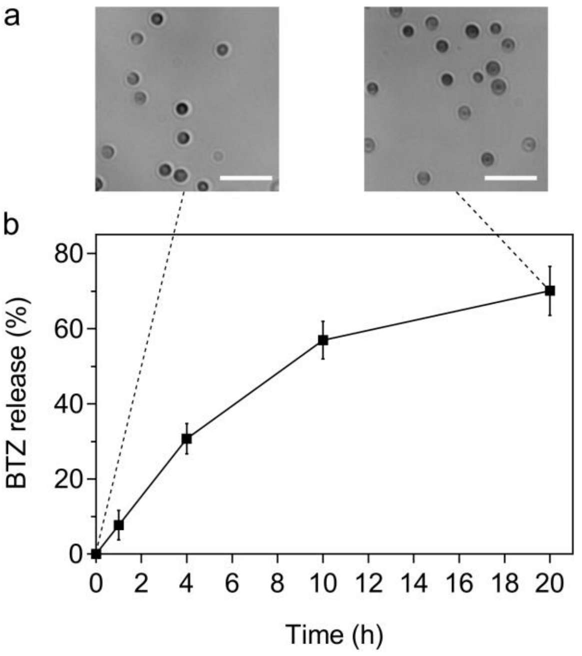

Figure S12. (a) DIC microscopy images of BTZ-conjugated (Fe^{III}-TA)$_1$ capsules before and after incubation for 20 h at pH 6.6. Scale bars are 5 µm. (b) Release of BTZ from (Fe^{III}-TA)$_1$ capsules over incubation for 20 h at pH 6.6.

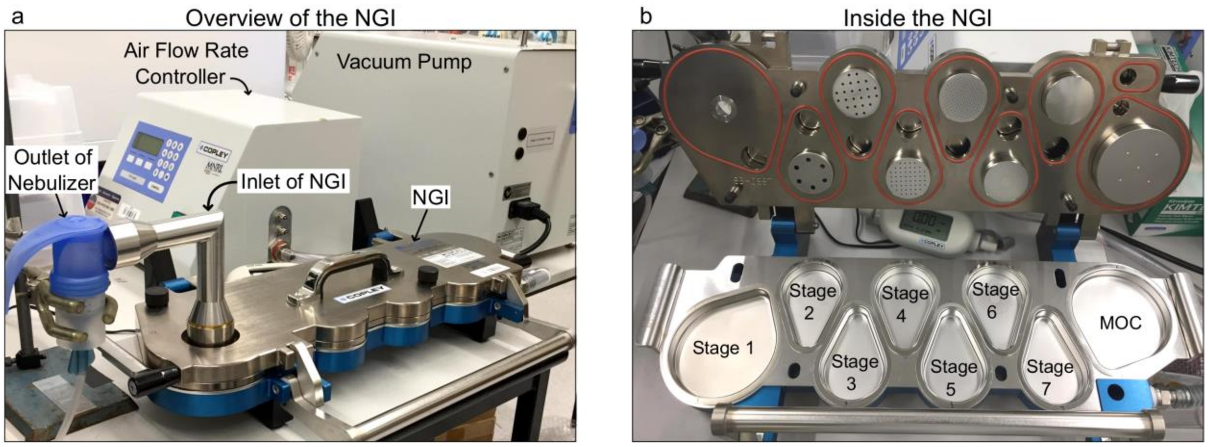

Figure S13. (a) Overview of the NGI setup showing the PARI jet nebulizer at the inlet "throat" of the NGI. The aerosols travel in air at the flow rate determined by the controller and the pump, through the main body of the NGI. (b) Interior of the NGI showing the different impactor stages (Stages 1–7) or collection cups that predict regional deposition in the lung. A micro-orifice collector (MOC) collects ultrafine droplets.

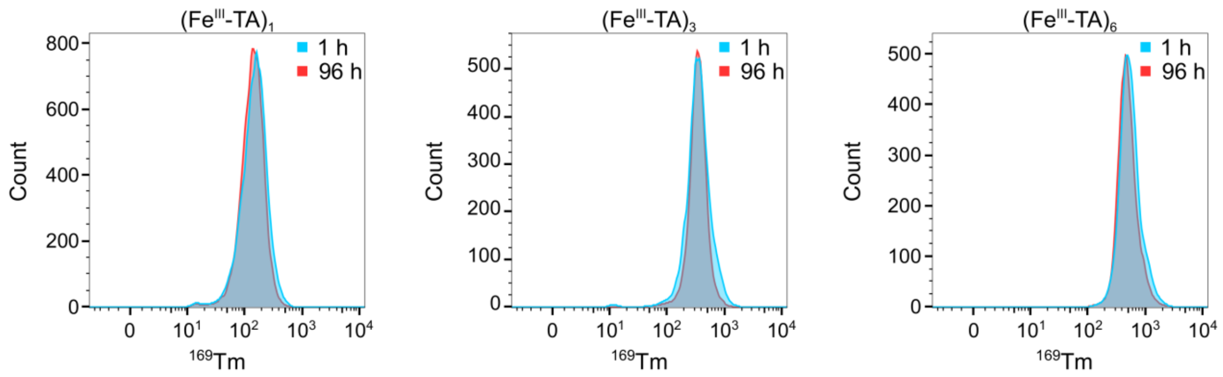

Figure S14. Overlay of mass cytometry histograms of Tm-labeled $(Fe^{III}\text{-TA})_1$, $(Fe^{III}\text{-TA})_3$, and $(Fe^{III}\text{-TA})_6$ capsules comparing the Tm signal intensity after incubation in water for 1 and 96 h.

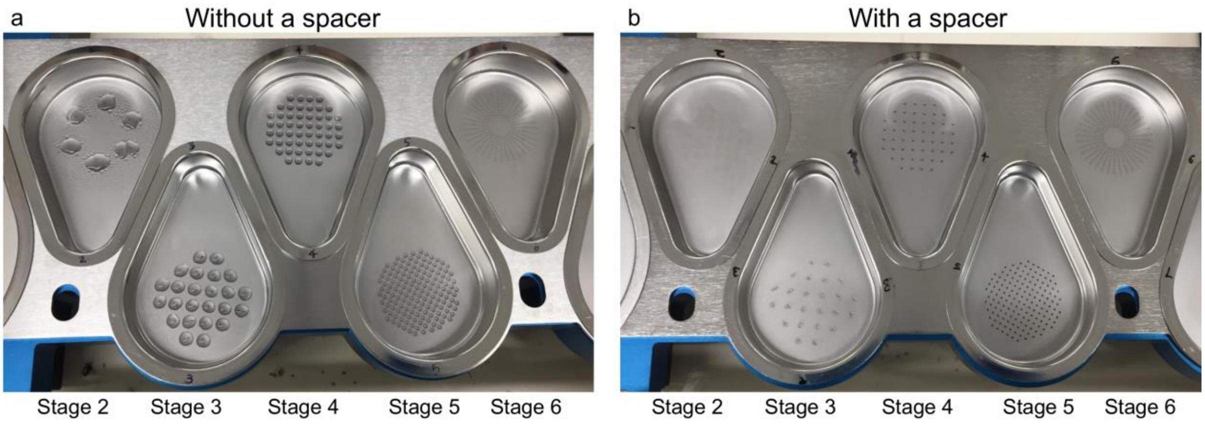

Figure S15. Sample deposition in the collection cups of the NGI after the nebulized aerosols (from $(Fe^{III}\text{-TA})_6$ capsule suspension) are (a) directly flowed into the NGI or (b) passed through a spacer before entering the NGI. The NGI was pre-cooled at 5 °C before the experiment and the air flow rate was set as 15 L min^{-1}. Aerosols were drawn into the NGI for 20 min before analyzing the collection cups. The temperature and relative humidity of ambient air were 25 °C and 32–40%, respectively.

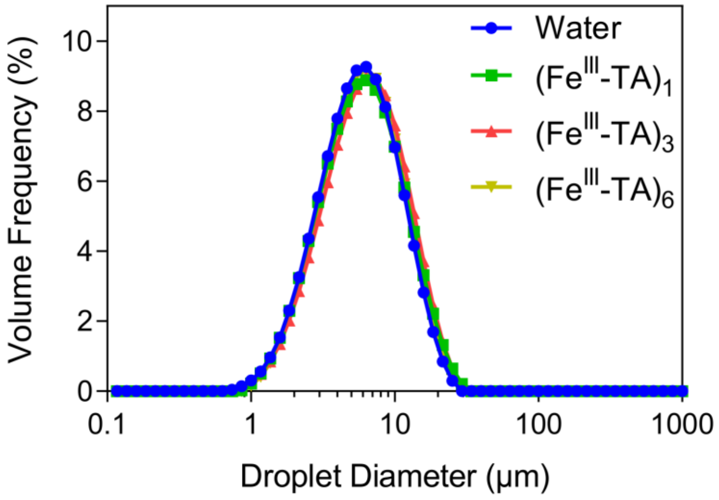

Figure S16. Aerosol size distribution of nebulized water, and $(Fe^{III}\text{-}TA)_1$, $(Fe^{III}\text{-}TA)_3$, and $(Fe^{III}\text{-}TA)_6$ capsule suspensions (1×10^5 capsules μL^{-1}) using a PARI air-jet nebulizer as evaluated by laser diffraction (Spraytec) at a flow rate of 15 L min^{-1} at 25 °C.

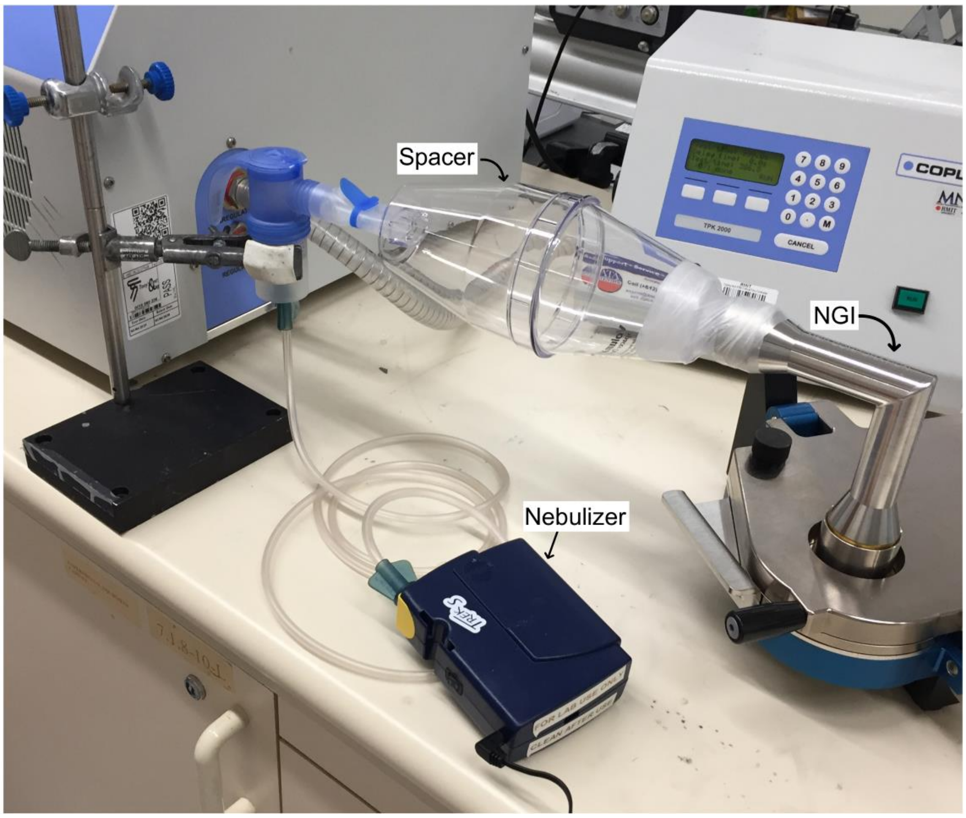

Figure S17. Photograph showing a spacer connected between the outlet of the nebulizer and the inlet of the NGI. The joint between the spacer and the inlet of the NGI was sealed with parafilm.

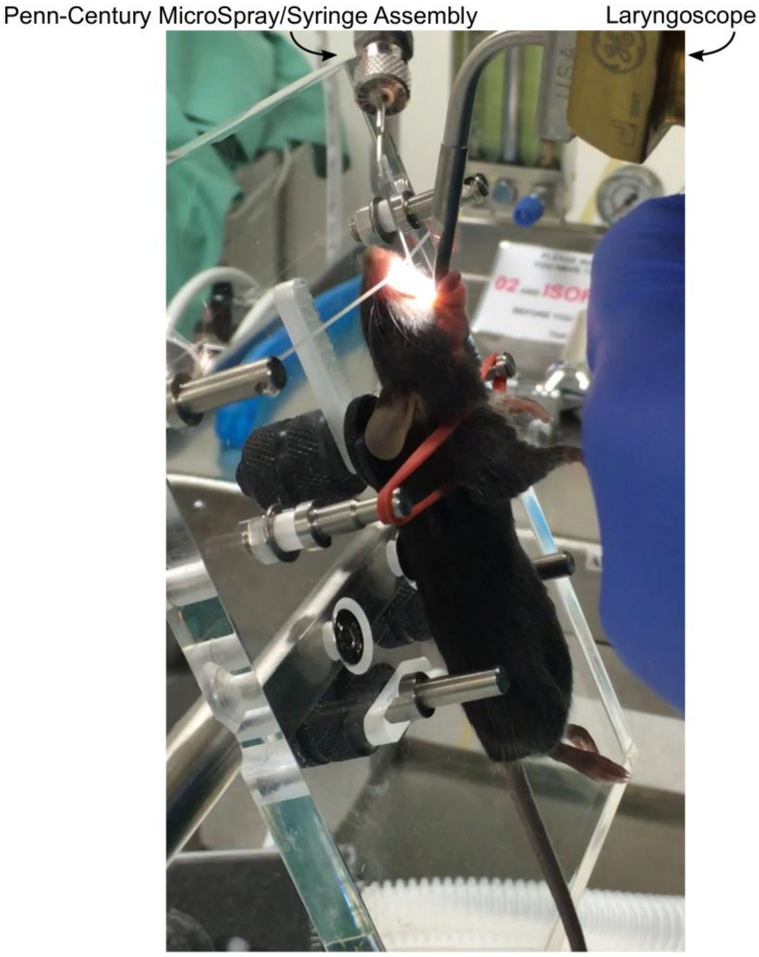

Figure S18. Photograph of a mouse being intratracheally administered with capsules using a Penn-Century microspray/syringe assembly under the guidance of a laryngoscope.

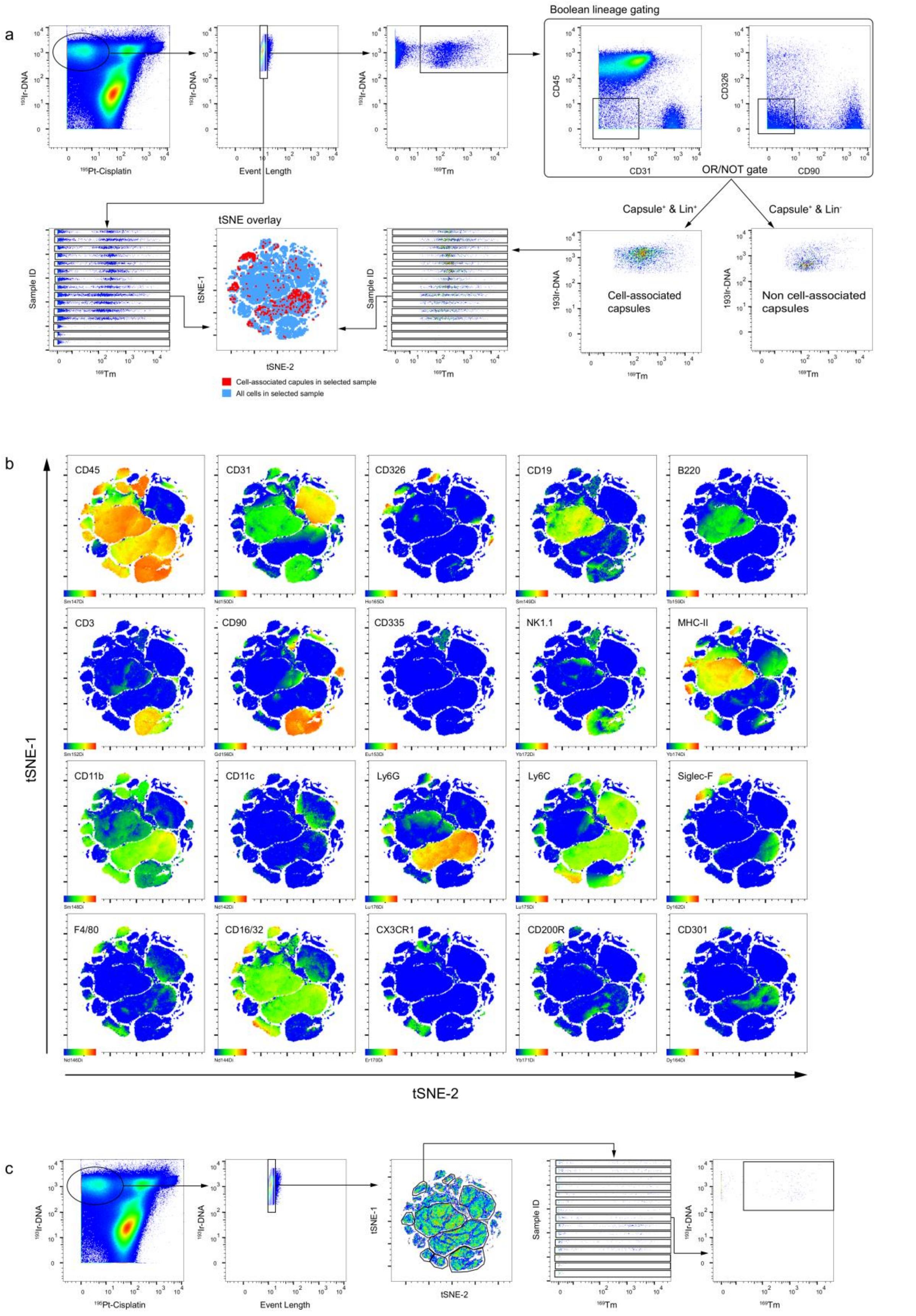
a
Boolean lineage gating
OR/NOT gate
Capsule⁺ & Lin⁺
Capsule⁺ & Lin⁻
Cell-associated capsules
Non cell-associated capsules
tSNE overlay
Cell-associated capules in selected sample
All cells in selected sample
Sample ID
tSNE-1
tSNE-2
Event Length
CD45
CD31
CD326
CD90
b
CD45
CD31
CD326
CD19
B220
CD3
CD90
CD335
NK1.1
MHC-II
CD11b
CD11c
Ly6G
Ly6C
Siglec-F
F4/80
CD16/32
CX3CR1
CD200R
CD301
tSNE-1
tSNE-2
c
Event Length
tSNE-1
tSNE-2
Sample ID

Figure S19. Analysis of in vivo mass cytometry data. (a) Mass cytometry data from all experimental animals were concatenated, and DNA^+ ($^{193}Ir^+$), live ($Cisplatin^-$) singlet ($Event\text{-}Length^{lo}$) events were selected. *t*-SNE was performed on the concatenated file, and non-cell-associated capsules were identified concurrently using Boolean gating. Events from individual animals were then selected and cells that had associated capsule signals were overlaid on the *t*-SNE plot from the corresponding animal. (b) Expression intensity of cell markers in the *t*-SNE plot of the concatenated sample. Not all markers are shown. (c) Gating of cell populations from specific experimental animals for determination of capsule-associated signals. In the plot shown, alveolar macrophages were selected.

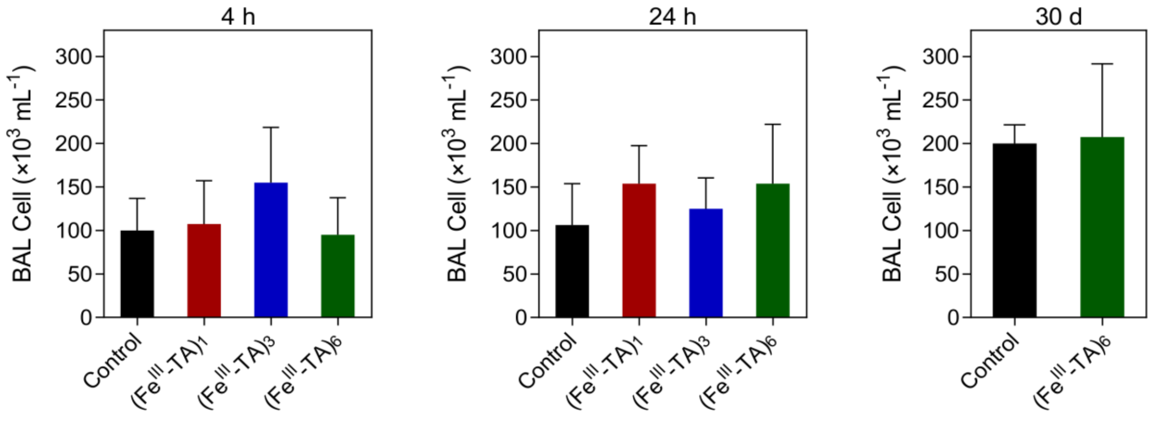

Figure S20. Total number of BAL cells in the lungs of mice at 4 h, 24 h, and 30 days post-intratracheal administration of DPBS (control), $(Fe^{III}\text{-TA})_1$, $(Fe^{III}\text{-TA})_3$, or $(Fe^{III}\text{-TA})_6$ capsules. The data are presented as the mean ± SD (four mice per group); no significant differences were observed between control and $(Fe^{III}\text{-TA})_1$, $(Fe^{III}\text{-TA})_3$, or $(Fe^{III}\text{-TA})_6$ group (one-way ANOVA with Tukey's multiple comparisons test).

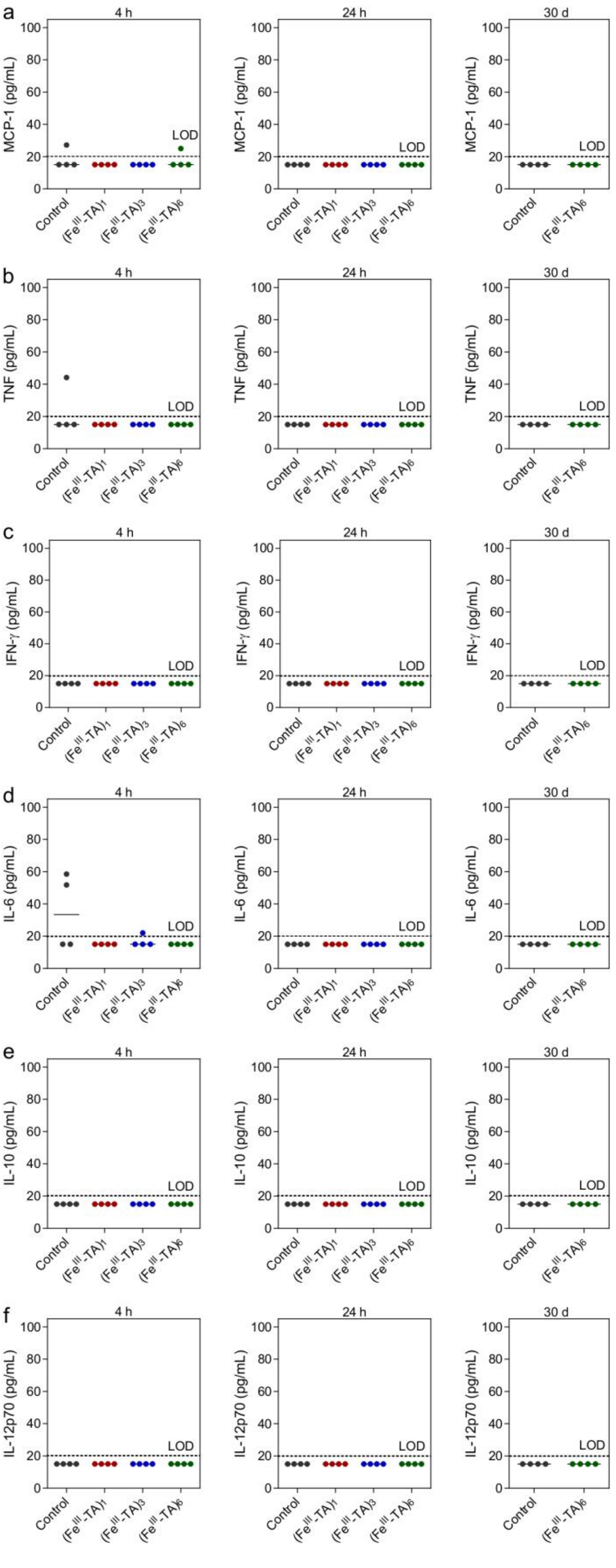

Figure S21. Cytokine levels in BALF of mice at 4 h, 24 h, and 30 days post-intratracheal administration of DPBS (control), $(Fe^{III}\text{-TA})_1$, $(Fe^{III}\text{-TA})_3$, or $(Fe^{III}\text{-TA})_6$ capsules: (a) MCP-1,

(b) TNF, (c) IFN-γ, (d) IL-6, (e) IL-10, and (f) IL-12p70. The dashed line represents the limit of detection (LOD, 20 pg mL^{-1}) of the assay. The data are presented as the mean ± SD (four mice per group).

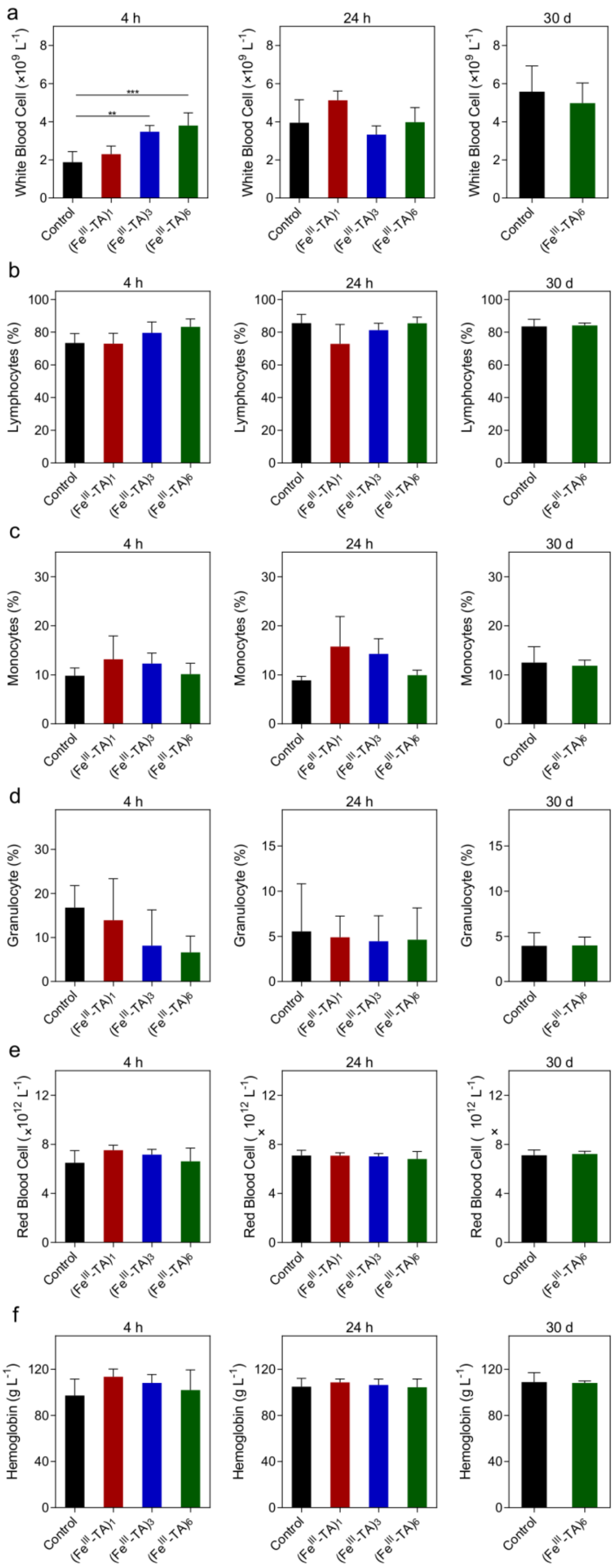

Figure S22. Effect of capsule administration on hematological parameters of mice at 4 h, 24 h, and 30 days post-intratracheal administration of DPBS (control), (Fe^{III}-TA)$_1$, (Fe^{III}-TA)$_3$, or

$(Fe^{III}\text{-}TA)_6$ capsules. (a) Number of white blood cells. Percentage of (b) lymphocytes, (c) monocytes, and (d) granulocytes with respect to the total white blood cells. (e) Number of red blood cells. (f) Concentration of hemoglobin. Fresh blood taken from mice was analyzed by the HEMAVET blood analysis system. Data are presented as the mean ± SD (four mice per group); *** in (a) 4 h indicates $p < 0.001$ control *vs*. $(Fe^{III}\text{-}TA)_6$, whereas ** in (a) 4 h indicates $p < 0.01$ control *vs*. $(Fe^{III}\text{-}TA)_3$; no significant differences were observed between the control and $(Fe^{III}\text{-}TA)_1$, $(Fe^{III}\text{-}TA)_3$, or $(Fe^{III}\text{-}TA)_6$ group in all other data sets (one-way ANOVA with Tukey's multiple comparisons test).

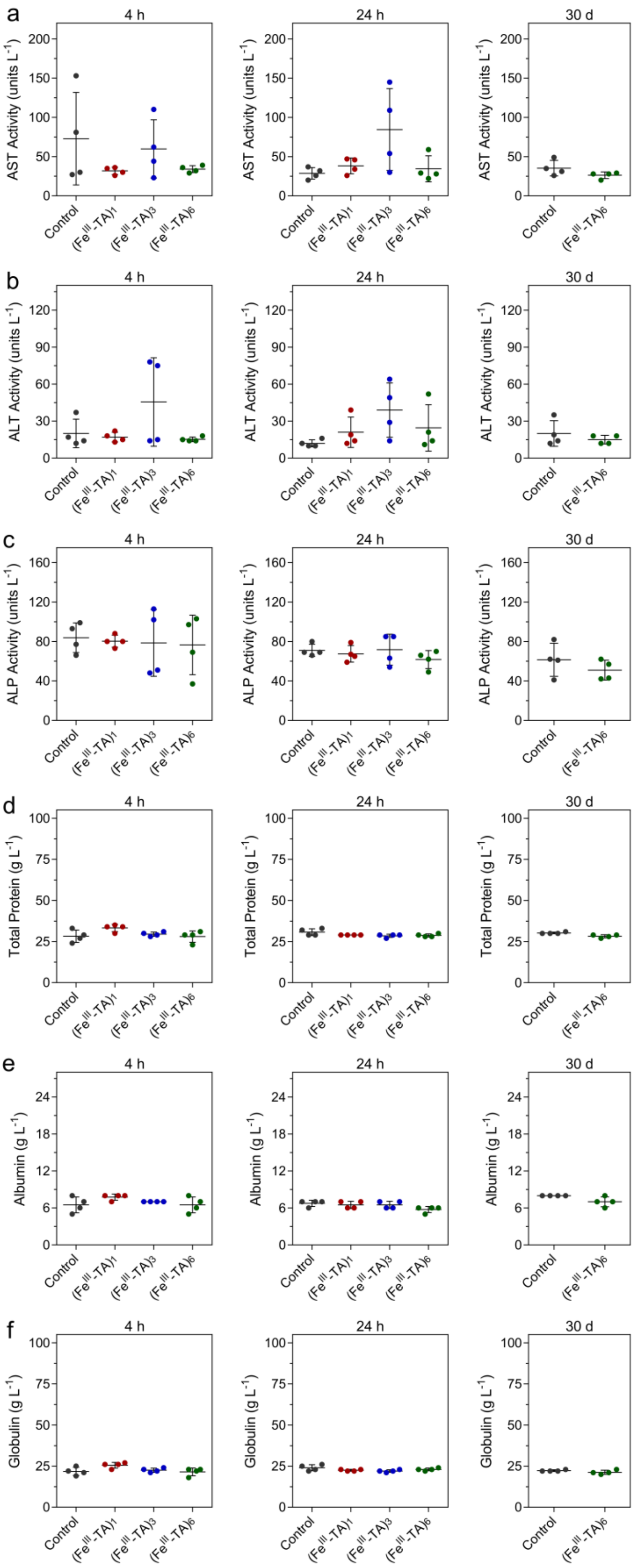

Figure S23. Liver toxicity study of mice at 4 h, 24 h, and 30 days post-intratracheal administration of DPBS (control), (Fe^{III}-TA)$_1$, (Fe^{III}-TA)$_3$, or (Fe^{III}-TA)$_6$ capsules. Serum

levels of liver enzymes: (a) aspartate aminotransferase (AST), (b) alanine aminotransferase (ALT), and (c) alkaline phosphatase (ALP). Concentration of (d) total protein, (e) albumin, and (f) globulin in serum. No significant differences were observed between the control and $(Fe^{III}\text{-}TA)_1$, $(Fe^{III}\text{-}TA)_3$, or $(Fe^{III}\text{-}TA)_6$ group in all data sets (one-way ANOVA with Tukey's multiple comparisons test).

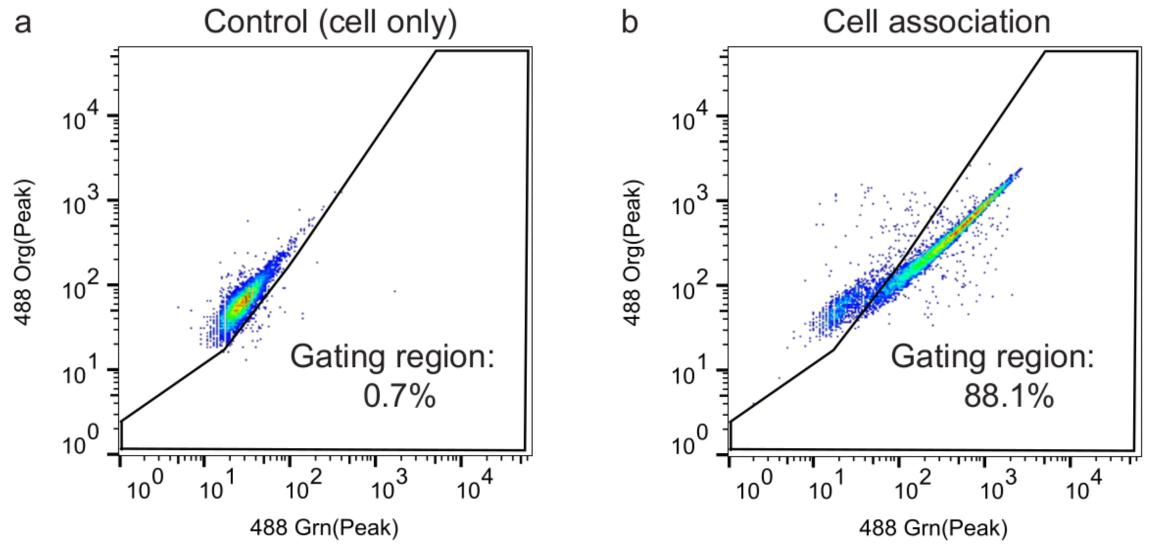

Figure S24. Gating strategy for cell association analysis of capsules using flow cytometry (A50-Micro Apogee Flow Systems). Cytograms were plotted with the 488 Orange channel (emission 560–590 nm) against the 488 Green channel (emission 500–540 nm). The same gating region was applied for (a) untreated and (b) treated A549 cells (treated cells were incubated with capsules for designated periods of time). The capsules were fluorescently labeled by loading them with dextran–fluorescein isothiocyanate. Cells that displayed stronger fluorescence (488 Green) intensity than untreated cells were identified as those associated with the capsules.

Table S1. Physical Properties of the $(Fe^{III}\text{-}TA)_1$, $(Fe^{III}\text{-}TA)_3$, and $(Fe^{III}\text{-}TA)_6$ Capsules[a]

Capsule	$(Fe^{III}\text{-}TA)_1$	$(Fe^{III}\text{-}TA)_3$	$(Fe^{III}\text{-}TA)_6$
Shell thickness (nm)	107.6 ± 30.4	161.7 ± 30.7	206.6 ± 31.7
Stiffness (mN m^{-1})	414.0 ± 130.4	636.9 ± 152.8	870.6 ± 257.8
ζ-potential (mV)	−40 ± 4	−35 ± 4	−35 ± 4
Diameter (μm) in aqueous solution	1.3 ± 0.1	1.4 ± 0.1	1.5 ± 0.1
Diameter (μm) dried in air	0.9 ± 0.1	1.0 ± 0.1	1.1 ± 0.1
Mass (pg)	0.4 ± 0.1	1.1 ± 0.1	2.2 ± 0.2
Density (g cm^{-3})	1.1 ± 0.3	2.1 ± 0.2	3.2 ± 0.3

[a]Shell thickness was determined from TEM (n = 100); stiffness was determined from colloidal-probe AFM (n = 15); ζ-potential was measured in phosphate buffer (2 mM, pH 7.4) by microelectrophoresis (n = 3); capsule diameters in aqueous solution and dried in air were determined from DIC microscopy and TEM (n = 30), respectively. Mass was determined by weighing a known number of freeze-dried capsules. Density was obtained by dividing mass by volume, where volume was calculated based on capsule diameter in air with the assumption that the capsule has a spherical structure.

Table S2. Aerodynamic Diameter of the $(Fe^{III}\text{-}TA)_1$, $(Fe^{III}\text{-}TA)_3$, and $(Fe^{III}\text{-}TA)_6$ Capsules[a]

Capsule	$(Fe^{III}\text{-}TA)_1$	$(Fe^{III}\text{-}TA)_3$	$(Fe^{III}\text{-}TA)_6$
MMAD without a spacer (μm)	6.20 ± 0.32	6.10 ± 0.27	6.24 ± 0.36
MMAD with a spacer (μm)	1.31 ± 0.07	1.85 ± 0.10	2.54 ± 0.20
Estimated D_a of dry capsules (μm)	0.94	1.45	1.96

[a]MMAD was determined based on the NGI distribution and using an algorithm based on ISO 27427 (Nebulizing Systems and Components) via mmadcalculator.com. Estimated D_a was calculated based on Equation 1 (main manuscript) using the average capsule density and diameter (air dried) in Table S1.

Table S3. Details of the Antibodies Used in the Mass Cytometry Study

Target	Label	Clone	Supplier	Primary/Secondary
CD11c	^{142}Nd	N418	In-house	Primary
CD64	^{143}Nd	X54-5/7.1	In-house	Primary
CD16/32	^{144}Nd	93	In-house	Primary
CD4	^{145}Nd	RM4-5	Fluidigm	Primary
F4/80	^{146}Nd	BM8	In-house	Primary
CD45	^{147}Sm	30-F11	In-house	Primary
CD11b	^{148}Nd	M1/70	In-house	Primary
CD19	^{149}Sm	6D5	In-house	Primary
CD3e	^{152}Sm	145-2C11	In-house	Primary
CD335	^{153}Eu	29A1.4	In-house	Primary
CD90.2	^{156}Gd	30-H12	In-house	Primary
B220	^{159}Tb	RA3-6B2	In-house	Primary
Siglec-F	^{162}Dy	E50-2440	In-house	Primary
CD301	^{164}Dy	LOM14	In-house	Primary
CD326	^{165}Ho	G8.8	Fluidigm	Primary
TIM4	^{166}Er	RMT4-54	In-house	Primary
CD209b	^{167}Er	22D1	In-house	Primary
CD8	^{168}Er	53-6.7	Fluidigm	Primary
CX3CR1	^{170}Er	SA011F11	In-house	Primary
NK1.1	^{172}Yb	PK136	In-house	Primary
MHCII	^{174}Yb	M5/114.15.2	In-house	Primary
Ly6C	^{175}Lu	HK1.4	In-house	Primary
Ly6G	^{176}Yb	1A8	In-house	Primary
CD31	Biotin	390	Ebioscience	Primary
anti-biotin	^{150}Nd	1D4-C5	Fluidigm	Secondary

References

[S1] J. L. Hutter, J. Bechhoefer, *Rev. Sci. Instrum.* **1993**, *64*, 1868.

[S2] A. Fery, F. Dubreuil, H. Möhwald, *New J. Phys.* **2004**, *6*, 18.

[S3] J. D. Berry, S. Mettu, R. R. Dagastine, *Soft Matter* **2017**, *13*, 1943.

[S4] M. Faria, M. Björnmalm, K. J. Thurecht, S. J. Kent, R. G. Parton, M. Kavallaris, A. P. R. Johnston, J. J. Gooding, S. R. Corrie, B. J. Boyd, P. Thordarson, A. K. Whittaker, M. M. Stevens, C. A. Prestidge, C. J. H. Porter, W. J. Parak, T. P. Davis, E. J. Crampin, F. Caruso, *Nat. Nanotechnol.* **2018**, *13*, 777.

Checklist

Minimum Information Reporting in Bio–Nano Experimental Literature

The MIRIBEL guidelines were introduced here: https://doi.org/10.1038/s41565-018-0246-4

The development of these guidelines was led by the ARC Centre of Excellence in Convergent Bio-Nano Science and Technology: https://www.cbns.org.au/. Any updates or revisions to this document will be made available here: http://doi.org/10.17605/OSF.IO/SMVTF. This document is made available under a CC-BY 4.0 license: https://creativecommons.org/licenses/by/4.0/.

The MIRIBEL guidelines were developed to facilitate reporting and dissemination of research in bio–nano science. Their development was inspired by various similar efforts:

- MIAME (microarray experiments): *Nat. Genet.* **29** (2001), 365; http://doi.org/10.1038/ng1201-365
- MIRIAM (biochemical models): *Nat. Biotechnol.* **23** (2005) 1509; http://doi.org/10.1038/nbt1156
- MIBBI (biology/biomedicine): *Nat. Biotechnol.* **26** (2008) 889; http://doi.org/10.1038/nbt.1411
- MIGS (genome sequencing): *Nat. Biotechnol.* **26** (2008) 541; http://doi.org/10.1038/nbt1360
- MIQE (quantitative PCR): *Clin. Chem.* **55** (2009) 611; http://doi.org/10.1373/clinchem.2008.112797
- ARRIVE (animal research): *PLOS Biol.* **8** (2010) e1000412; http://doi.org/10.1371/journal.pbio.1000412
- *Nature*'s reporting standards:
  - Life science: https://www.nature.com/authors/policies/reporting.pdf; e.g., *Nat. Nanotechnol.* **9** (2014) 949; http://doi.org/10.1038/nnano.2014.287
  - Solar cells: https://www.nature.com/authors/policies/solarchecklist.pdf; e.g., *Nat. Photonics* **9** (2015) 703; http://doi.org/10.1038/nphoton.2015.233
  - Lasers: https://www.nature.com/authors/policies/laserchecklist.pdf; e.g., *Nat. Photonics* **11** (2017) 139; http://doi.org/10.1038/nphoton.2017.28
- The "TOP guidelines": e.g., *Science* **352** (2016) 1147; http://doi.org/10.1126/science.aag2359

Similar to many of the efforts listed above, the parameters included in this checklist are **not** intended to be definitive requirements; instead they are intended as 'points to be considered', with authors themselves deciding which parameters are—and which are not—appropriate for their specific study.

This document is intended to be a living document, which we propose is revisited and amended annually by interested members of the community, who are encouraged to contact the authors of this document. Parts of this document were developed at the annual International Nanomedicine Conference in Sydney, Australia: http://www.oznanomed.org/, which will continue to act as a venue for their review and development, and interested members of the community are encouraged to attend.

After filling out the following pages, this checklist document can be attached as a "Supporting Information" document during submission of a manuscript to inform Editors and Reviewers (and eventually readers) that all points of MIRIBEL have been considered.

Supplementary Table 1. Material characterization*

Question	Yes	No
1.1 Are "**best reporting practices**" **available** for the nanomaterial used? For examples, see *Chem. Mater.* **28** (2016) 3535; http://doi.org/10.1021/acs.chemmater.6b01854 and *Chem. Mater.* **29** (2017) 1; http://doi.org/10.1021/acs.chemmater.6b05235		N/A
1.2 If they are available, **are they used**? If not available, ignore this question and proceed to the next one.		
1.3 Are extensive and clear instructions reported detailing all steps of **synthesis** and the resulting **composition** of the nanomaterial? For examples, see *Chem. Mater.* **26** (2014) 1765; http://doi.org/10.1021/cm500632c, and *Chem. Mater.* **26** (2014) 2211; http://doi.org/10.1021/cm5010449. Extensive use of photos, images, and videos are strongly encouraged. For example, see *Chem. Mater.* **28** (2016) 8441; http://doi.org/10.1021/acs.chemmater.6b04639	✓	
1.4 Is the **size** (or **dimensions**, if non-spherical) and **shape of** the nanomaterial reported?	✓	
1.5 Is the **size dispersity** or **aggregation** of the nanomaterial reported?	✓	
1.6 Is the **zeta potential** of the nanomaterial reported?	✓	
1.7 Is the **density (mass/volume)** of the nanomaterial reported?	✓	
1.8 Is the amount of any **drug loaded** reported? 'Drug' here broadly refers to functional cargos (e.g., proteins, small molecules, nucleic acids).	✓	
1.9 Is the **targeting performance** of the nanomaterial reported, including **amount** of ligand bound to the nanomaterial if the material has been functionalised through addition of targeting ligands?		N/A
1.10 Is the **label signal** per nanomaterial/particle reported? For example, fluorescence signal per particle for fluorescently labelled nanomaterials.	✓	
1.11 If a material property not listed here is varied, has it been **quantified**?	✓	
1.12 Were characterizations performed in a **fluid mimicking biological conditions**?		N/A
1.13 Are details of how these parameters were **measured/estimated** provided?	✓	
Explanation for **No** (if needed):<br>1.8 The relative amount of cargo that remained encapsulated in the capsules before and after nebulization was quantified.		

*Ideally, material characterization should be performed in the same biological environment as that in which the study will be conducted. For example, for cell culture studies with nanoparticles, characterization steps would ideally be performed on nanoparticles dispersed in cell culture media. If this is not possible, then characteristics of the dispersant used (e.g., pH, ionic strength) should mimic as much as possible the biological environment being studied.

Supplementary Table 2. Biological characterization*

Question	Yes	No
2.1 Are **cell seeding details**, including **number of cells plated, confluency at start of experiment,** and **time between seeding and experiment** reported?	✓	
2.2 If a standardised cell line is used, are the **designation and source** provided?	✓	
2.3 Is the **passage number** (total number of times a cell culture has been subcultured) known and reported?	✓	
2.4 Is the last instance of **verification of cell line** reported? If no verification has been performed, is the time passed and passage number since acquisition from trusted source (e.g., ATCC or ECACC) reported? For information, see *Science* **347** (2015) 938; http://doi.org/10.1126/science.347.6225.938		✓
2.5 Are the results from **mycoplasma testing** of cell cultures reported?	✓	
2.6 Is the **background signal of cells/tissue** reported? (E.g., the fluorescence signal of cells without particles in the case of a flow cytometry experiment.)	✓	
2.7 Are **toxicity studies** provided to demonstrate that the material has the expected toxicity, and that the experimental protocol followed does not?	✓	
2.8 Are details of media preparation (**type of media, serum,** any **added antibiotics**) provided?	✓	
2.9 Is a **justification of the biological model** used provided? For examples for cancer models, see *Cancer Res.* **75** (2015) 4016; http://doi.org/10.1158/0008-5472.CAN-15-1558, and *Mol. Ther.* **20** (2012) 882; http://doi.org/10.1038/mt.2012.73, and *ACS Nano* **11** (2017) 9594; http://doi.org/10.1021/acsnano.7b04855	✓	
2.10 Is characterization of the **biological fluid** (*ex vivo*/*in vitro*) reported? For example, when investigating protein adsorption onto nanoparticles dispersed in blood serum, pertinent aspects of the blood serum should be characterised (e.g., protein concentrations and differences between donors used in study).		N/A
2.11 For **animal experiments**, are the ARRIVE guidelines followed? For details, see *PLOS Biol.* **8** (2010) e1000412; http://doi.org/10.1371/journal.pbio.1000412	✓	
Explanation for **No** (if needed):<br>2.4 Cells were purchased from ATCC. The passage number is reported.<br>2.11 The animal experiments were approved by the Alfred Medical Research and Education Precinct (AMREP) Animal Ethics Committee (E/1625/2016/M). The details of experiments are provided in the experiment section.		

*For *in vitro* experiments (e.g., cell culture), *ex vivo* experiments (e.g., in blood samples), and *in vivo* experiments (e.g., animal models). The questions above that are appropriate depend on the type of experiment conducted.

Supplementary Table 3. Experimental details*

Question	Yes	No
3.1 For cell culture experiments: are **cell culture dimensions** including **type of well**, **volume of added media**, reported? Are cell types (i.e.; adherent vs suspension) and **orientation** (if non-standard) reported?	✓	
3.2 Is the **dose of material administered** reported? This is typically provided in nanomaterial mass, volume, number, or surface area added. Is sufficient information reported so that regardless of which one is provided, the other dosage metrics can be calculated (i.e. using the dimensions and density of the nanomaterial)?	✓	
3.3 For each type of imaging performed, are details of how **imaging** was performed provided, including details of **shielding, non-uniform image processing**, and any **contrast agents** added?	✓	
3.4 Are details of how the dose was administered provided, including **method of administration, injection location, rate of administration,** and details of **multiple injections**?	✓	
3.5 Is the methodology used to **equalise dosage** provided?	✓	
3.6 Is the **delivered dose** to tissues and/or organs (in vivo) reported, as % injected dose per gram of tissue (%ID g^{-1})?	✓	
3.7 Is **mass of each organ/tissue measured** and **mass of material** reported?		✓
3.8 Are the **signals of cells/tissues with nanomaterials** reported? For instance, for fluorescently labelled nanoparticles, the total number of particles per cell or the fluorescence intensity of particles + cells, at each assessed timepoint.	✓	
3.9 Are **data analysis details**, including **code used** for analysis provided?	✓	
3.10 Is the **raw data** or **distribution of values** underlying the reported results provided? For examples, see *R. Soc. Open Sci.* **3** (2016) 150547; http://doi.org/10.1098/rsos.150547, https://opennessinitiative.org/making-your-data-public/, http://journals.plos.org/plosone/s/data-availability, and https://www.nature.com/sdata/policies/repositories		✓
Explanation for **No** (if needed):<br>3.7 Mass of each organ was measured and biodistribution is reported as percentage of injected dose per gram of tissue (%ID g^{-1}).<br>3.10 Analysis of *in vitro* flow cytometry data and *in vivo* mass cytometry data is included in SI (Figure S11 and Figure S13). Raw data is available upon request.		

* The use of protocol repositories (e.g., *Protocol Exchange* http://www.nature.com/protocolexchange/) and published standard methods and protocols (e.g., *Chem. Mater.* **29** (2017) 1; http://doi.org/10.1021/acs.chemmater.6b05235, and *Chem. Mater.* **29** (2017) 475; http://doi.org/10.1021/acs.chemmater.6b05481) are encouraged.
